# Pellet Printing for Soft Robotic Devices

**DOI:** 10.1002/advs.202524237

**Published:** 2026-03-23

**Authors:** Yijia Wu, Ju‐Hung Chen, Ariana Olivares, Katherine Kostak, Stefan Pedicone, Savita V. Kendre, Markus P. Nemitz

**Affiliations:** ^1^ Department of Mechanical Engineering Tufts University Medford USA

**Keywords:** 3D printing, additive manufacturing, fused granulate fabrication, soft robots, thermoplastic elastomers

## Abstract

Rapid prototyping of soft robotic devices is often constrained by manual fabrication or additive manufacturing methods that are limited in material choice or require extensive post‐processing. Fused Granulate Fabrication offers a scalable alternative by extruding thermoplastic pellets through a screw‐based extruder, enabling continuous, high‐throughput printing and access to a broad range of commercially available materials, from rigid plastics to silicone‐soft elastomers with Shore hardness as low as 6A. Reliable 3D printing of airtight pneumatic soft structures at volumetric flow rates up to 5 mm^3^/s is demonstrated by addressing inconsistent extrusion and stringing issues through a combination of hardware optimization and a materials‐centered printing strategy. Extrusion and oozing tests are used to construct material‐specific oozing performance profiles, establishing practical guidelines for material selection in FGF printing, and are linked to key rheological descriptors. The mechanical performance of thermoplastic styrenic block copolymer pellets is characterized, revealing Mullins‐effect‐induced softening, and fabricated pneumatic actuators exhibit durability exceeding 100 000 bending cycles. Demonstrations include a pneumatically actuated robotic hand, a multi‐chamber robotic fish, and a soft pressure cuff. FGF enables the digital fabrication of large‐scale, airtight soft robotic devices using commercially available thermoplastic pellets, providing a versatile, cost‐effective, and scalable alternative to soft lithography with mechanical performance comparable to silicone elastomers.

## Graphical abstract

Fused Granulate Fabrication (FGF) is established here as a reliable method for fabricating soft, airtight robotic devices. Through coordinated optimization of hardware, material selection, and process parameters, this approach enables high‐speed printing of thermoplastic elastomers with silicone‐like softness and modulus. Systematic studies of extrusion behavior and mechanical performance define a quantitative workflow that supports scalable, high‐throughput manufacturing of soft robotic devices.

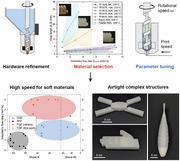

## 1 Introduction

Soft robotics is a transformative field that enables the creation of adaptable, compliant, and biomimetic systems [1], with applications spanning medical devices [2], assistive wearables [3], robotic locomotion [4], and manipulation [5], among others. The intrinsic deformability of soft materials enhances safety in human‐robot interaction [6], improves resilience to mechanical impact [7], and allows robots to change their shape [8], thereby enabling functionalities beyond the reach of traditional robots with rigid body plans. Despite these advances, the fabrication of soft systems, particularly those requiring airtight properties and materials within the Shore 00 hardness range, remains a major challenge. Soft lithography has long been used to produce such systems, but it involves laborious fabrication steps and depends heavily on operator skill, limiting scalability, reproducibility, and broader adoption beyond specialized laboratories [9]. As a result, many state‐of‐the‐art demonstrations, including multi‐gait soft robots [8], bistable valves [10], and soft grippers [11], still rely on custom molds, extensive post‐processing, and manual assembly.

Digital fabrication techniques, including Fused Filament Fabrication (FFF), Direct Ink Writing (DIW), Stereolithography (SLA), Material Jetting (MJ), and Selective Laser Sintering (SLS), have been explored as mold‐free alternatives to soft lithography for fabricating soft robotic devices. However, each method presents fundamental trade‐offs in material compatibility, geometric fidelity, and printing reliability that have limited their widespread use [12, 13, 14]. While FFF remains popular due to its low cost and accessibility [15, 16, 17, 18, 19, 20, 21, 22, 23], its performance is constrained by filament mechanics. Commercial soft filaments are typically thermoplastic polyurethanes (TPUs) with Shore hardness ≥ 60A, which are relatively stiff compared to silicones and often lead to extrusion issues such as buckling and jamming during printing [24]. DIW is a versatile technique compatible with a wide range of soft materials, such as silicones, hydrogels, and liquid‐crystal elastomers, and particularly suitable for exploring new formulations. However, DIW generally requires careful rheological tuning to achieve printability, and commercial materials or materials formulated by non‐experts often suffer from flow instabilities or demand long, process‐sensitive curing steps [25, 26, 27, 28, 29, 30]. SLA and MJ offer high print resolution but rely on brittle photopolymers with viscoelastic material characteristics, poor fatigue resistance, and UV degradation [31, 32, 33]. SLS avoids support structures but is limited by challenges in powder removal and a narrow range of available soft elastomers (≥ 40A). Although these techniques have enabled important advances in soft device manufacturing, their material and process constraints and availability continue to limit the geometric complexity, mechanical performance, and reproducibility of fabricated devices. These challenges become more severe in fluidically driven soft systems, where airtightness and geometric precision are essential for integrating channels and enclosed chambers within a single build. SLA, MJ, SLS, and DIW struggle with internal cavities where residual resin, powder, or ink becomes trapped, especially in long, narrow channels and complex internal networks [34, 35]. Although MJ supports the use of support materials, their removal still hinders the fabrication of fully enclosed chambers. FFF methods mitigate this issue but introduce their own challenges, including leakage and limited print reliability for complex geometries. 3D printing airtight structures reliably requires slow print speeds (<20 mm/s) and restrictive design rules, such as multi‐layered walls [18] or Euler‐path toolpaths to minimize interfacial voids [22]. Other strategies to improve airtightness include gravity‐assisted layer sealing [36] and vision‐based closed‐loop control for real‐time defect correction [24]. Although FFF has become more reliable for fabricating soft robotic devices, it remains fundamentally constrained in material selection, as it is largely limited to materials with higher Shore hardness and cannot access the low Shore hardness typical of silicones.

A promising alternative to established printing strategies is Fused Granulate Fabrication (FGF). FGF has been primarily used in industry rather than in research laboratories. It offers several industry relevant advantages, including high throughput, low material cost, broad material availability, existing expertise from injection molding, and the capability to print at large scales. FGF processes raw pellets through an extrusion screw, achieving higher extrusion rates and access to a wider range of thermoplastics than FFF [37, 38]. Thermoplastic styrenic block copolymers (TPS, or TPE‐S) can achieve lower Shore hardness values than other classes of thermoplastic elastomers (TPEs) [39]. Previous studies have demonstrated the feasibility of desktop FGF systems for printing conductive pellets [39, 40, 41], ultra‐soft membranes in the Shore 00–30 range [42], and other specialized materials [43, 44]. However, these demonstrations have largely focused on proof‐of‐concept geometries or individual materials. The systematic evaluation of material‐dependent extrusion stability, transient oozing behavior, and long‐duration fabrication reliability for complex, fluidically driven soft robotic devices is still lacking. Consequently, although thousands of thermoplastic pellets offer mechanical properties suitable for soft systems, their compatibility with desktop FGF printing remains largely unexplored.

A key barrier to broader adoption is the mismatch between pellet formulations and the requirements of additive manufacturing. Most thermoplastic pellets are optimized for injection molding or continuous extrusion, not layer‐by‐layer deposition. In screw‐based extrusion, residual pressure relaxation, melt elasticity, and pellet compression lead to inconsistent flow and severe stringing, particularly for soft materials. Hardware mitigation strategies such as cooling zones [42] or mechanical needle valves [45] reduce these effects but increase system complexity and remain insufficiently validated across materials. Even on identical hardware, material‐dependent rheology strongly governs extrusion stability and residual flow, indicating that material selection is as critical as hardware design. In FFF and DIW, printability is commonly linked to rheological descriptors such as yield stress, zero shear viscosity, and shear thinning behavior [46, 47, 48]. These approaches were developed for filament feedstocks or paste‐like inks and do not capture the transient post‐extrusion flow dynamics specific to screw‐driven pellet extrusion. For soft robotic systems, printability must also account for airtightness, long‐duration structural stability, and mechanical reliability under cyclic loading. Existing screening methods, therefore, cannot be directly adopted to pellet‐based fabrication of soft systems, motivating the development of an application‐specific evaluation methodology.

In this study, we systematically evaluate the suitability of commercially available TPS pellets for pellet‐based fabrication of soft robotic devices by quantifying extrusion throughput, transient oozing behavior, rheological properties, print quality, and mechanical performance. We identify the dominant failure modes that limit airtight fabrication and demonstrate material and process strategies to mitigate them. Rather than relying primarily on hardware modifications, we adopt a material‐informed methodology that links extrusion stability and residual flow to measurable rheological descriptors, enabling the definition of practical operating windows for reliable pellet‐based printing. To validate this approach, we characterize the mechanical properties of printed TPS structures through tensile testing and cyclic loading, quantify Mullins effect‐induced stress softening, and assess the bending response and fatigue durability of pneumatic actuators. Using optimized materials and parameters, we demonstrate long‐duration fabrication up to 24 hours and large‐scale printing of airtight, fluidically driven soft robotic devices, including a multi‐segment soft robotic hand, an articulated robotic fish, and a wearable pressure cuff. Together, these results establish FGF as a practical and scalable platform for rapid prototyping of soft robotic systems using commercially available thermoplastic pellets spanning Shore hardness values from 6A to 50A.

## 2 Pellet Printing With Soft Materials

**FIGURE 1:**
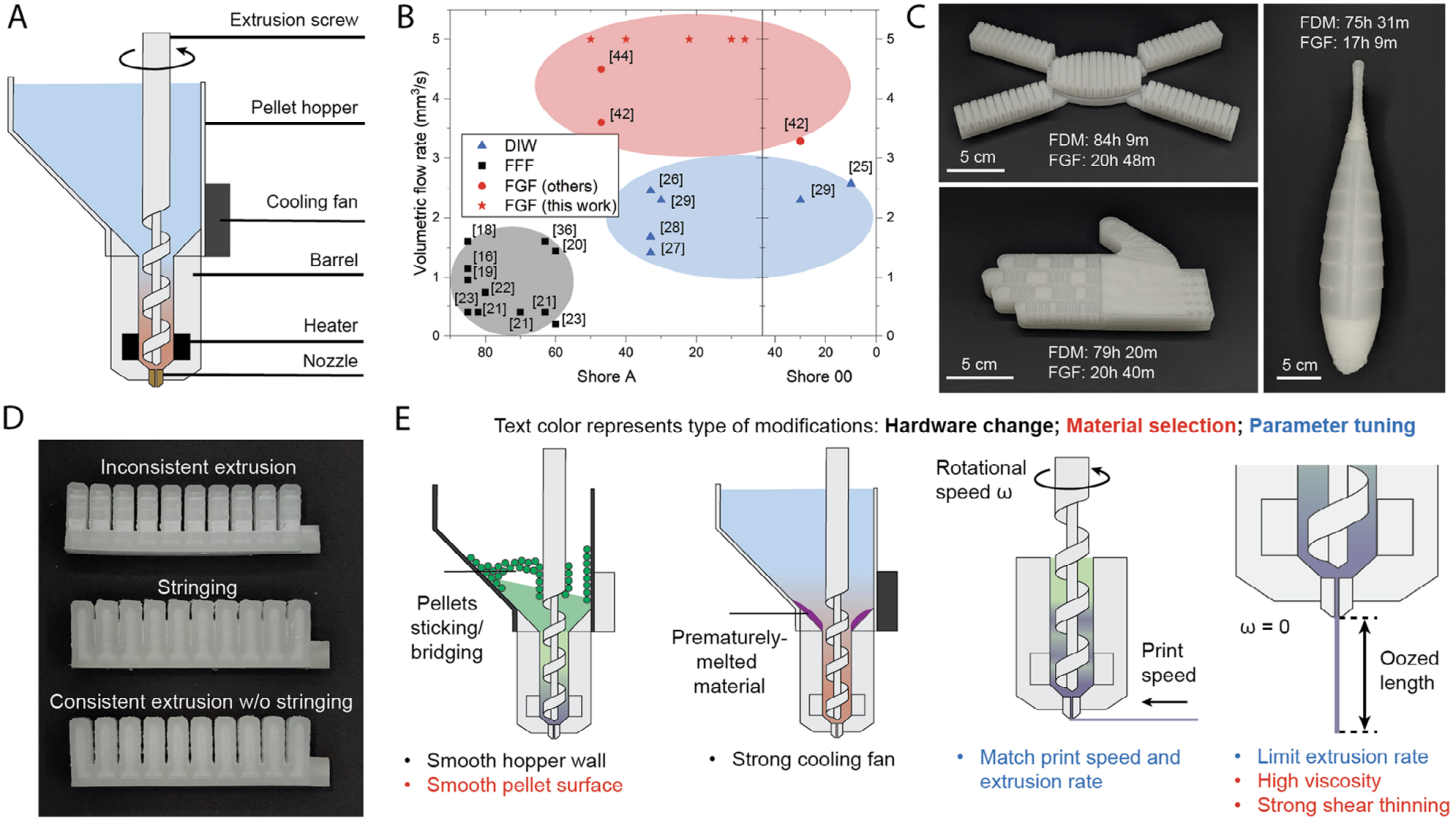
**Overview of the Fused Granulate Fabrication (FGF) printing process**. (A) Schematic of pellet extruder. (B) Estimated volumetric flow rate versus Shore A hardness from prior FFF and FGF studies on airtight soft robot fabrication, highlighting that FGF achieves higher volumetric flow rates while supporting softer materials than FFF. Only studies with nozzle diameters in the range of 0.3‐0.6 mm are included to ensure comparability, as nozzle size strongly influences both resolution and volumetric flow rate. (C) Example of large‐scale complex geometries printed with FGF, demonstrating that FGF significantly accelerates the fabrication of large soft structures. Print parameters are summarized in Supporting Information, Table S2. (D) Images of printed pneumatic actuators highlighting two common defects, inconsistent extrusion and stringing, that can compromise functionality, alongside an optimized print demonstrating successful mitigation through tuning. (E) Causes and solutions we implemented for inconsistent extrusion and stringing issues.

FGF, or pellet printing, operates on the same layer‐by‐layer deposition principle as FFF but uses thermoplastic pellets instead of filament feedstock. Pellets are fed by gravity or pneumatics into a screw‐driven extruder, where they are melted and deposited through a heated nozzle (Figure 1A). This screw‐based extrusion decouples material rigidity from processability, enabling the reliable printing of soft thermoplastic elastomers with stiffness comparable to silicones. In contrast, filament‐fed FFF systems are prone to buckling when processing soft materials. To quantify this advantage, we analyzed published studies reporting airtight soft robotic components fabricated via DIW, FFF, or FGF. Volumetric flow rates $Q\;[{\mathrm{mm}}^{3}/s]$ were estimated from reported nozzle diameters d[mm], layer heights h[mm], and print speeds v[mm/s] using their relation Q=d×h×v, assuming the extrusion width equals the nozzle diameter. The resulting comparison of volumetric flow rate versus material Shore hardness shows that FGF consistently achieves higher flow rates, even for softer materials (Figure 1B). FGF reduced the fabrication time of our demonstrators from several days using FFF to a single day (Figure 1C).

While FGF offers several advantages over FFF, including higher flow rates and broader compatibility with soft materials, it remains challenging to achieve consistent, high‐quality prints. To promote the broader adoption of FGF for fabricating soft robotic devices, we used a commercially available pellet extruder, a standard 3D printer, and commercially available soft thermoplastic pellets in our experiments. Although small, flat structures can be fabricated reliably, longer print durations and increased structural complexity lead to recurring issues such as mid‐print failures, leakage, and stringing. We identified two main causes of these defects: inconsistent extrusion and oozing (Figure 1D). Severe occurrences of either can result in complete print failure, while milder cases compromise airtightness and device performance. Overcoming these challenges requires a coordinated strategy combining hardware modifications, material selection, and process parameter optimization (Figure 1E).

### 2.1 Enabling Consistent Extrusion

Inconsistent extrusion leads to non‐uniform wall thickness, which can cause leakage in under‐extruded regions or require higher actuation pressures in over‐extruded areas of the printed soft device. We mitigated this issue through a three‐step approach addressing feeding, melting, and extrusion: (1) improving pellet feeding by redesigning the print head hopper with smoother walls and selecting pellets with low surface friction to avoid pellet bridging; (2) preventing premature melting by enhancing active cooling using a higher performing fan; and (3) calibrating extrusion flow to match print speed, ensuring consistent material deposition. Further details on these improvements are provided in the Supporting Information Section 2.1.

### 2.2 Reducing Stringing

Stringing refers to the formation of unintended thin strands of thermoplastic between printed features, caused by residual oozing during the non‐print travel of the print head. Even after the extrusion screw stops rotating, molten material can continue to ooze due to gravity and residual back pressure within the barrel. This effect is especially pronounced in soft, elastic materials and can lead to geometric distortions, internal voids, and ultimately leakage or reduced device performance. In FFF, stringing is typically mitigated through filament retraction; however, applying retraction to elastomers often introduces extrusion instability, buckling, or inconsistent flow, making it unreliable for printing compliant materials [24]. Another widely used strategy is reducing the nozzle temperature to increase melt viscosity. While effective in suppressing oozing, this approach simultaneously reduces the achievable volumetric flow rate and print speed, and can significantly weaken interlayer adhesion due to insufficient thermal bonding [49]. As a result, these process‐based strategies typically suppress stringing at the expense of print stability, throughput, or structural integrity.

An alternative strategy is to suppress stringing through intrinsic material rheology rather than process‐based mitigation [46, 48]. FGF enables this approach by allowing direct use of pelletized elastomers with widely varying viscoelastic properties. In this work, we find that materials combining high low‐shear viscosity, strong shear‐thinning behavior during extrusion, and predominantly elastic response at high frequency exhibit substantially reduced residual oozing, enabling clean, high‐speed printing without compromising extrusion stability or interlayer adhesion.

### 2.3 Print Quality

To evaluate the print quality achievable with the optimized FGF process, we evaluated dimensional accuracy and unsupported feature printability. Dimensional accuracy was quantified along the X, Y, and Z directions of 20 mm calibration cubes using mean signed error, root‐mean‐square error (RMSE), and standard deviation across 6 cubes. Stiffer materials (22A–50A) exhibited low RMSE values (<0.15 mm) and minimal systematic bias, while softer materials showed increased dimensional deviations, reflecting the trade‐off between achievable softness and geometric fidelity (Table S5, Supporting Information). Printability of unsupported features was further evaluated through bridging and overhang tests. The critical and maximum bridge lengths and overhang angles increase with material stiffness (Table S6, Supporting Information), with stiffer pellets supporting longer bridges (up to 70 mm) and steeper overhangs (up to 25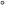), consistent with their higher melt strength.

## 3 Material Screening Strategy to Minimize Oozing

Reliable pellet‐based fabrication of soft devices requires materials that suppress residual oozing to minimize stringing while maintaining high extrusion flow rates. We evaluated commercially available thermoplastic elastomer pellets spanning Shore hardness values from 6A to 63A. While all tested materials produced airtight pneumatic actuators under controlled conditions, certain grades, such as Filaflex 60A and Baiyu 30A, exhibited excessive residual oozing during travel moves, which increased the risk of unintended material deposition and air leakage in complex geometries. To address this limitation, we combined extrusion characterization with rheological analysis to establish a material screening methodology for FGF‐based soft device fabrication.

### 3.1 Extrusion and Oozing Characteristics

**FIGURE 2:**
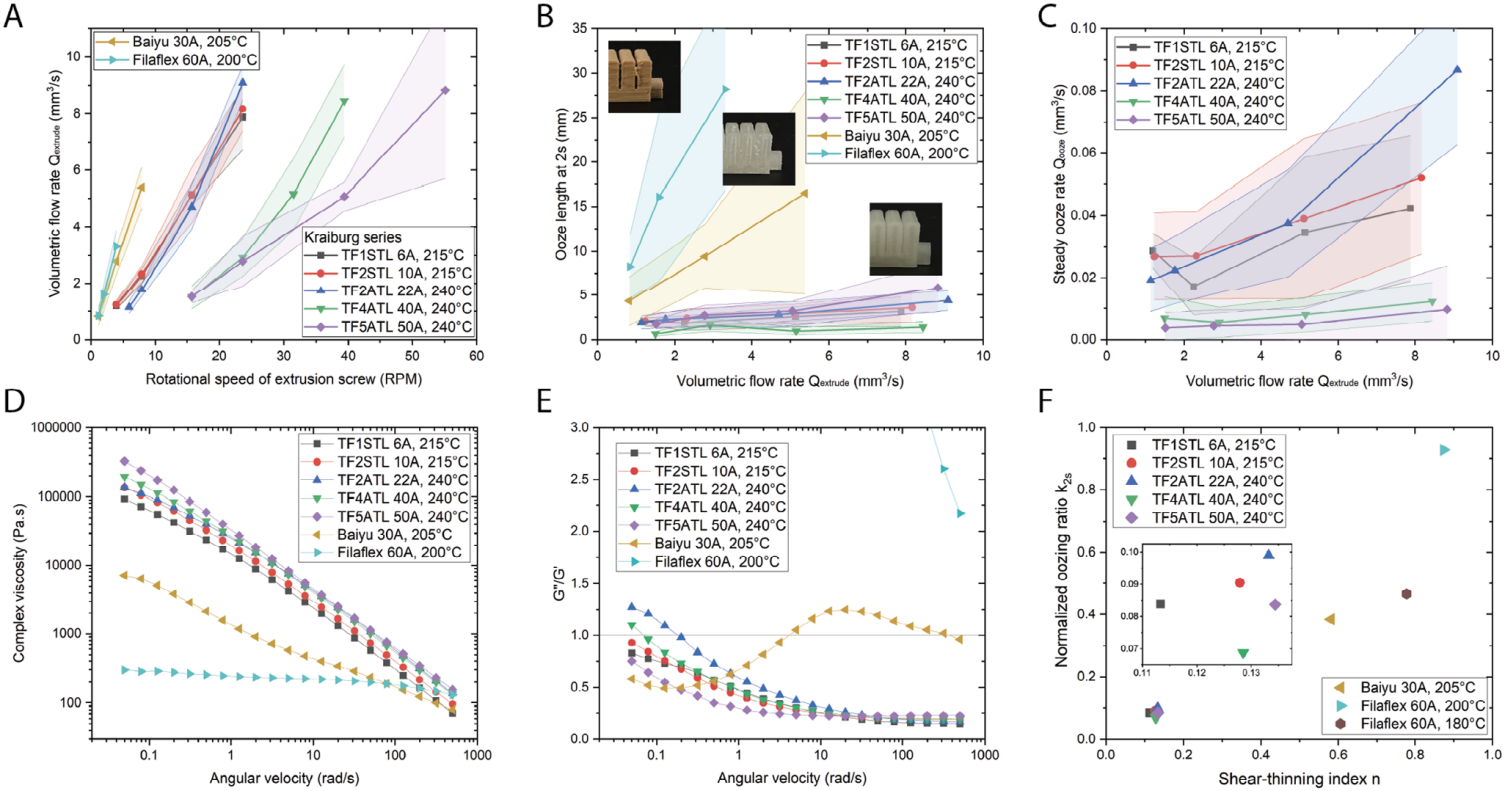
**Extrusion and rheology characterization of TPS pellets**. (A) Volumetric flow rate as a function of rotational speed of the extrusion screw for different pellets at their respective print temperatures, with the shaded error band showing the 95% confidence intervals from ten tests. (B) Oozed length after 2 seconds plotted against volumetric extrusion flow rate for different materials at their respective printing temperatures. At the same extrusion flow rate, materials with high initial oozing exhibit excessive stringing and unwanted material deposition during travel moves, whereas the selected materials (6A, 10A, 22A, 40A, 50A) show minimal stringing under comparable conditions. (C) Steady ooze rate as a function of extrusion flow rate, indicating that while initial oozing rates are comparable across materials, stiffer materials exhibit faster decay of residual flow. (D) Complex viscosity as a function of angular frequency for all tested materials. (E) G”/G' as a function of angular frequency for all tested materials. When G”/G'> 1, materials behave predominately viscous. When G”/G'<1, materials behave predominately elastic. (F) Normalized oozing rate for the first 2 seconds ${\bar{k}}_{2s}$ as a function of shear thinning index n, illustrating the relationship between rheological behavior and oozing performance. Materials with stronger shear‐thinning (lower n) exhibit reduced ooze rate, consistent with rapid viscosity recovery as shear rate decreases after extrusion cessation.

The extrusion flow rate ($Q_{\text{extrude}}$) increased approximately linearly with the rotational speed of the extrusion screw, consistent with drag‐flow‐dominated screw extrusion behavior (Figure 2A). Within the tested Kraiburg TPE grades, 6A, 10A, 22A, 40A, and 50A, stiffer materials required higher screw rotational speeds than softer grades to achieve the same volumetric flow rate and exhibited greater variability in flow response. At a fixed extrusion flow rate, significant material‐dependent differences emerged after extrusion ceased (Figure S4, Movie S1, Supporting Information). We quantified oozing performance using the oozed length within the first 2 seconds ($L_{2s}$), corresponding to typical short travel‐move durations. Plotting $L_{2s}$ against extrusion flow rate yielded a material‐specific oozing performance profile (Figure 2B), revealing the inherent tradeoff between high throughput and low residual flow. For the Kraiburg TPE materials, $L_{2s}$ remained below 3 mm at an extrusion rate of 3 ${\mathrm{mm}}^{3}$/s and below 3.5 mm at 5 ${\mathrm{mm}}^{3}$/s. All Kraiburg TPE grades could be printed without obvious stringing at 5 ${\mathrm{mm}}^{3}$/s, corresponding to a print speed of 50 mm/s under our print settings. In contrast, Baiyu 30A and Filaflex 60A exhibited substantially larger $L_{2s}$ values even at extrusion rates below 1 ${\mathrm{mm}}^{3}$/s (Table S7, Supporting Information). Representative printing results at 3 ${\mathrm{mm}}^{3}$/s for Filaflex 60A, Baiyu 30A, and TF5ATL 50A are also included in Figure 2B for comparison.

To facilitate a comparison across materials, operating conditions, and extrusion rates, we introduce a normalized oozing metric defined as $k=Q_{ooze}/Q_{extrude}$, where $Q_{ooze}$ is the oozing flow rate and $Q_{extrude}$ is the extrusion flow rate. Evaluating this metric over the first 2 seconds (${\bar{k}}_{2s}$) captures the initial transient oozing behavior. For example, Filaflex 60A at 200 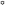 C exhibited a ${\bar{k}}_{2s}$ of 0.93, indicating that the material flow remained nearly continuous immediately after extrusion ceased, and all Kraiburg TPE materials exhibited ${\bar{k}}_{2s}$ less than 0.1. Calculating k over the interval from 15 to 30 seconds characterizes longer‐timescale residual flow behavior. Within the Kraiburg TPE series, softer grades (6A, 10A, 22A) exhibited a slower decay in oozing rate after screw rotation ceased (Figure 2C).

### 3.2 Rheological Characterization

We performed oscillatory frequency sweeps to characterize the complex viscosity η, storage modulus $G^{\prime }$, and loss modulus $G^{\prime \prime }$ of each material at the selected temperatures. The angular frequency refers to the oscillatory deformation frequency applied in the rheometer and serves as a proxy for shear rate $\dot{\gamma }$ through the Cox–Merz relation. It is not directly related to the rotational speed of the extrusion screw in the FGF system. For reference, the apparent shear rate during extrusion can be estimated from the volumetric flow rate Q using $\dot{\gamma }∼\frac{32Q}{\pi d_{n}^{3}}\frac{3n+1}{4n}$, where $d_{n}$ is the nozzle diameter and n is the shear‐thinning index [50]. Three rheological descriptors were extracted to interpret differences in oozing behavior: (i) low‐shear viscosity, (ii) shear‐thinning index n, and (iii) the $G^{\prime }/G^{\prime \prime }$ crossover frequency (Table S8, Supporting Information).

Materials with poor oozing resistance (30A, 60A) exhibited lower low‐shear viscosity, weaker shear‐thinning behavior, and more pronounced viscous response (Figure 2D, E). A lower low‐shear viscosity reduces resistance to residual flow once active shear is removed, facilitating continued material discharge under internal pressure and gravity. A correlation between shear‐thinning index n and initial oozing ratio was observed across the full material set (Figure 2F), indicating that rapid viscosity recovery as shear decreases plays a key role in suppressing residual flow. However, within the Kraiburg TPE series, rheological differences alone do not fully account for variations in printability, indicating that additional factors, such as viscoelastic relaxation behavior and the mechanical properties of the pellets, also influence oozing performance.

Overall, our material screening strategy serves as a practical guideline for choosing materials that ooze less for high throughput. Successful FGF material candidates for airtight soft robotic devices should ideally exhibit high low‐shear viscosity to resist residual flow and strong shear‐thinning behavior to enable high extrusion throughput. A smooth pellet surface is also desired for consistent pellet feeding.

## 4 Material Characterization

**FIGURE 3:**
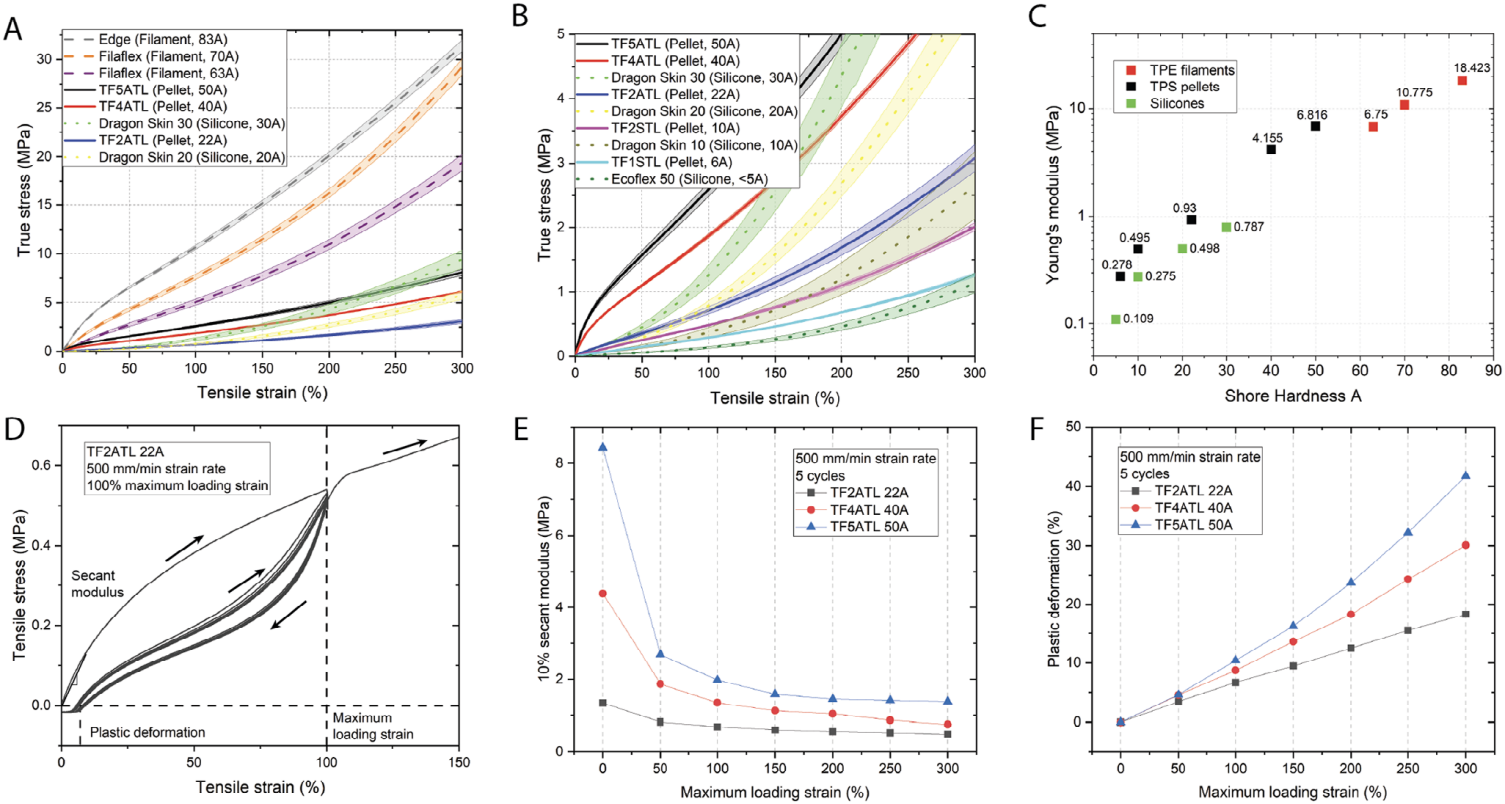
**Mechanical characterization of TPE filaments, TPS pellets, and silicone materials for soft structure fabrication**. (A) True stress–strain curves up to 300% strain, with shaded 95% confidence intervals from five tests. (B) Zoomed‐in view highlighting stress–strain response of materials with lower Young's modulus. (C) Young's modulus versus Shore hardness, showing the correlation between stiffness and hardness. (D) Representative tensile cyclic loading of 22A TPS pellets, illustrating loading–unloading hysteresis, definitions of maximum cycling strain, secant modulus, and plastic deformation. (E) Dependence of the 10% secant modulus on maximum cyclic strain for 22A, 40A, and 50A pellets, showing progressive stress softening (Mullins effect) with increasing strain amplitude. Stiffer materials exhibit a larger relative modulus reduction under identical maximum strain. (F) Dependence of plastic deformation on maximum cyclic strain for 22A, 40A, and 50A pellets. Stiffer materials exhibit greater accumulated permanent deformation under equivalent loading conditions.

To guide our material selection and learn the mechanical properties of printed structures, we characterized representative TPE filaments, TPS pellets, and silicone rubbers. Five dogbone specimens per material were prepared using FDM printing, FGF printing, and molding in accordance with ASTM D412 standard and tested under uniaxial tension. We converted the engineering strain to true strain to capture nonlinear behavior at low strain, and plotted stress‐strain curves up to 300% strain along with 95% confidence intervals for each material (Figure 3A). Our results show that TPE filaments exhibit high stiffness and strain softening within the first 100% strain, reflecting their high initial modulus and limited extensibility. In contrast, TPS pellets displayed substantially lower stiffness. Softer pellets, particularly those with Shore hardness below 22A, exhibited low modulus and minimal initial softening, showing mechanical behavior comparable to commercial silicones (Figure 3B). The correlation between Young's modulus and Shore hardness confirms the general trend of increasing stiffness with Shore hardness and demonstrates the wider modulus range achievable with TPS pellets (Figure 3C).

TPS is a compound of styrenic block copolymer (SBC), polyolefin, oil, additives, and fillers. For the materials evaluated in this study, polypropylene (PP) acts as the polyolefin component. The Mullins effect is characterized by stress softening that depends on the previously applied maximum strain [51]. Although it was first observed in filled rubbers, similar behavior has also been documented in unfilled and multiphase elastomeric systems [52]. To evaluate the Mullins effect in the TPS materials, we conducted cyclic tensile tests on 22A, 40A, and 50A pellets at a strain rate of 500 mm/min with incrementally increasing maximum loading strain. The loading–unloading curves reveal a reduction in secant modulus and the presence of plastic deformation (Figure 3D). The majority of mechanical softening occurs during the first loading cycle, after which subsequent cycles exhibit more stable behavior (Figure S6, Supporting Information).

Increasing the maximum loading strain leads to a monotonic decrease in the 10% secant modulus for all three materials, especially for the first 50% strain, and a corresponding increase in plastic deformation (Figure 3E, F). Across the tested materials, stiffer grades exhibited larger modulus reduction and greater accumulated plastic deformation under equivalent maximum strain. According to the material provider, within the same material series, stiffer grades (TF5ATL 50A) contain relatively higher PP and lower oil content compared to softer grades (TF2ATL 22A), which indicates compositional influence. However, in multiphase TPS systems, stress softening may arise from several mechanisms, including deformation or disruption of the thermoplastic PP phase, rearrangement of the SBC network, and interactions between oil, fillers, and polymer domains [52]. Identifying the primary microstructural origin of the Mullins effect was beyond the scope of the present work.

## 5 Case Study: Pellet Printed Pneumatic Actuators

### 5.1 Mechanical Characterization

**FIGURE 4:**
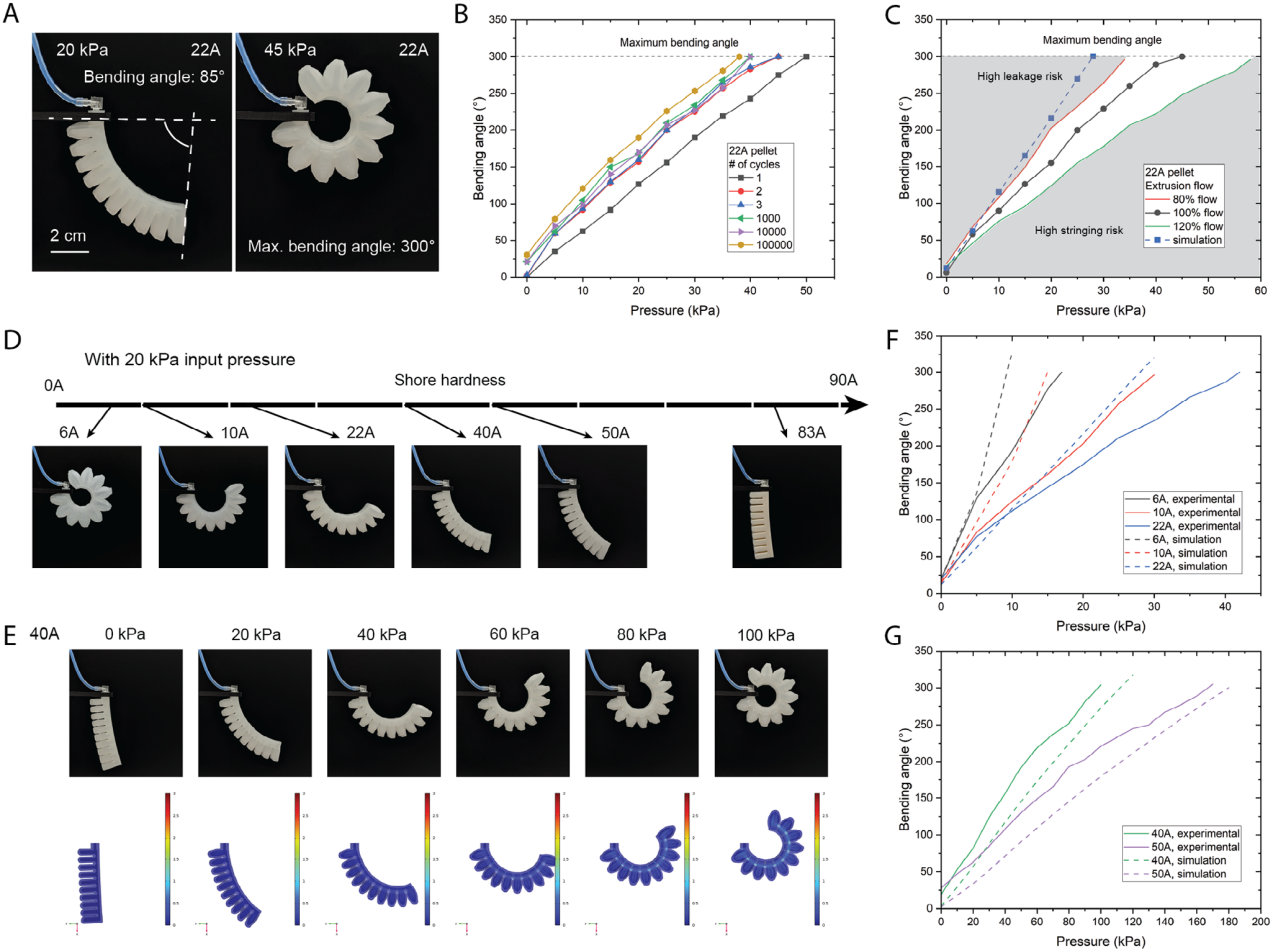
**Characterization of soft actuators printed with TPS pellets using FGF**. (A) PneuNet actuator fabricated from 22A pellets, illustrating bending angles of 85 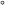 and 300 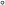 at maximum deformation. (B) Bending angle as a function of pressure measured over 1–100,000 actuation cycles, demonstrating stable and repeatable actuator performance. (C) Effect of extrusion flow rate on actuator bending behavior; shaded regions denote operating conditions associated with leakage and stringing. The flow percentage indicates the relative setting with respect to the recommended flow rate for the 22A pellet and does not represent the absolute volumetric flow rate. (D) Comparison of actuator bending at 20 kPa for materials with varying Shore hardness illustrating the influence of material stiffness on deformation performance. (E) Experimental and simulated deformation profiles of a 40A actuator under increasing pressure, highlighting the agreement between measured and predicted bending behavior. (F) Bending angle as a function of pressure for 6A, 10A, and 22A actuators, showing reduced experimental bending compared to simulations due to over‐extrusion effects during fabrication. (G) Comparison of bending angles for 40A and 50A actuators, with discrepancies between experimental and simulated results attributed to the Mullins effect and material stress softening.

To evaluate the performance of soft robotic devices fabricated via FGF, we designed, printed, and tested a pneumatic network (PneuNet) actuator using TPS pellets of varying Shore hardness [53]. Actuation performance was evaluated by measuring the bending angle as a function of input pressure (Figure 4A) and comparing the results with silicone‐molded and filament‐printed PneuNet actuators (Figure S7, Supporting Information).

To assess long‐term durability, we subjected the 22A actuator to continuous operation for up to 100,000 actuation cycles (Figure 4B). The pressure–bending relationship remained consistent throughout testing, showing minimal degradation in peak angle and response behavior. These results confirm that soft actuators fabricated via FGF maintain airtightness and mechanical integrity under extended use. A distinct change between the first and second actuation cycles reflects the Mullins effect, which stabilizes thereafter and does not compromise long‐term functionality. The Mullins effect observed in 3D‐printed TPS structures can be viewed as a form of mechanical conditioning, during which the actuator reaches its steady‐state performance.

We further examined the effect of extrusion flow rate on actuator performance (Figure 4C). Under‐extrusion resulted in poor interlayer adhesion and leakage, while over‐extrusion caused stringing and geometric distortion. By fine‐tuning the flow rate, we achieved reproducible fabrication of airtight actuators with consistent bending behavior. However, Finite Element Analysis (FEA) aligned more closely with the response of under‐extruded actuators, suggesting that the over‐extrusion typically used to ensure airtightness causes deviations between experimental and simulated performance.

We investigated the material‐dependent mechanical response of pneumatic actuators by fabricating PneuNets using FGF with TPS pellets spanning Shore hardness values from 6A to 50A (Figure 4D). Under identical input pressures, softer materials produced significantly greater deformation due to their lower elastic modulus and higher compliance. In contrast, a PneuNet printed via FFF using a commercial TPU filament (Ninjaflex Edge, 83A) exhibited minimal bending. This result highlights the importance of 3D printable materials with Shore hardness comparable to silicones, which here enable large deformations in soft pneumatic actuators. After repeated actuation cycles, stress softening and associated plastic deformation, consistent with the Mullins effect, produced residual curvature in the PneuNets in the unactuated, unpressurized state. The increase in pre‐bending angle from 22 to 50A aligns with the higher levels of plastic deformation quantified during cyclic tensile testing (Figure S7, Supporting Information).

### 5.2 Numerical Simulation

To assess actuator performance and evaluate simulation accuracy, we performed FEA using material models calibrated with uniaxial tensile test data. For each material, a hyperelastic constitutive model was fitted to capture nonlinear deformation, and the pressurization induced response of the PneuNet actuators was simulated. The predicted deformations were then compared with experimental measurements to quantify model fidelity. The simulated deformation profiles showed strong agreement with the experimentally observed bending shapes, confirming that the fitted material models accurately captured the overall deformation behavior (Figure 4E). However, systematic discrepancies were evident in the pressure–bending angle relationships across different materials.

For actuators fabricated from 6A, 10A, and 22A TPS pellets, the experimentally measured bending angles were consistently lower than predicted by the numerical model (Figure 4F). This deviation likely results from over‐extrusion during printing, which increased wall thickness and overall stiffness, effects not captured in the idealized model. In contrast, actuators printed with 40A and 50A pellets exhibited bending angles that exceeded the simulated predictions (Figure 4G). This deviation is attributed to the Mullins effect, a stress softening phenomenon not captured by the material models, which becomes more pronounced in stiffer elastomers under large strain.

These comparisons highlight the importance of studying fabrication‐induced variability and time‐dependent viscoelastic effects to improve the predictive accuracy of computational models for soft pneumatic actuators.

## 6 Demonstrations

### 6.1 Soft Robotic Hand

**FIGURE 5:**
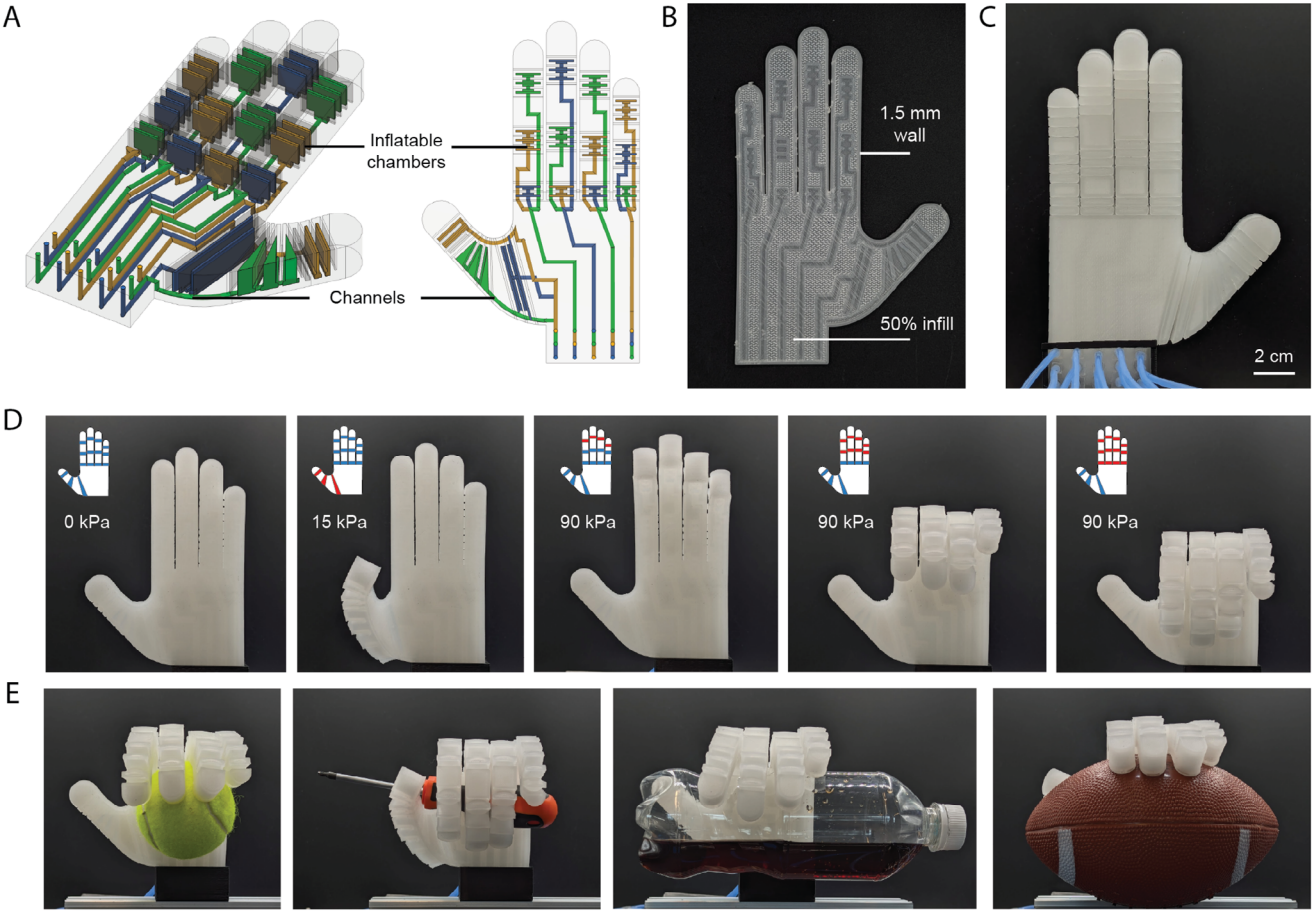
**Robotic hand demonstrating reliable FGF printing of a complex fluidic device**. (A) Semi‐transparent CAD rendering of the hand design, with blue, yellow, and green regions indicating internal channels and inflatable chambers. (B) Close‐up image of the printed hand paused at a height of 3 mm during fabrication, showing thin‐wall structures, internal channels, and 50% gyroid infill. (C) Image of the backside of the printed hand. (D) Actuation sequence illustrating the transition from the extended to fully grasping state through sequential activation of chambers in all five fingers. Hand schematics in the top left corner indicate actuator status, with red denoting actuated and blue representing unactuated chambers. (E) Demonstration of the robotic hand grasping a variety of everyday objects.

We designed, fabricated, and tested a fully soft robotic hand to demonstrate the capability of FGF to produce complex, integrated fluidic devices. Printing the robotic hand with 22A pellets required 20 hours and 40 minutes (Movie S2, Supporting Information). The hand consists of five fingers, each containing three independent embedded pneumatic chambers connected through integrated fluidic pathways (Figure 5A). The structure was printed monolithically, without post‐processing or assembly. Thin outer walls (1.5 mm, three perimeters) and a 50% gyroid infill were used to balance compliance and airtightness (Figure 5B). All inflatable chambers connect to internal channels that merge at the back of the hand within a compact routing manifold, allowing 15 individual tubes to deliver pressurized air to each chamber for actuation (Figure 5C). Sequential actuation of these chambers enables coordinated bending across the finger segments, culminating in a full grasping configuration (Figure 5D). The robotic hand successfully grasped objects of varying sizes and geometries (Figure 5E, Movie S3, Supporting Information), demonstrating the effectiveness of FGF in fabricating functional, monolithic soft robotic systems with intricate internal channel networks.

### 6.2 Soft Robotic Fish

**FIGURE 6:**
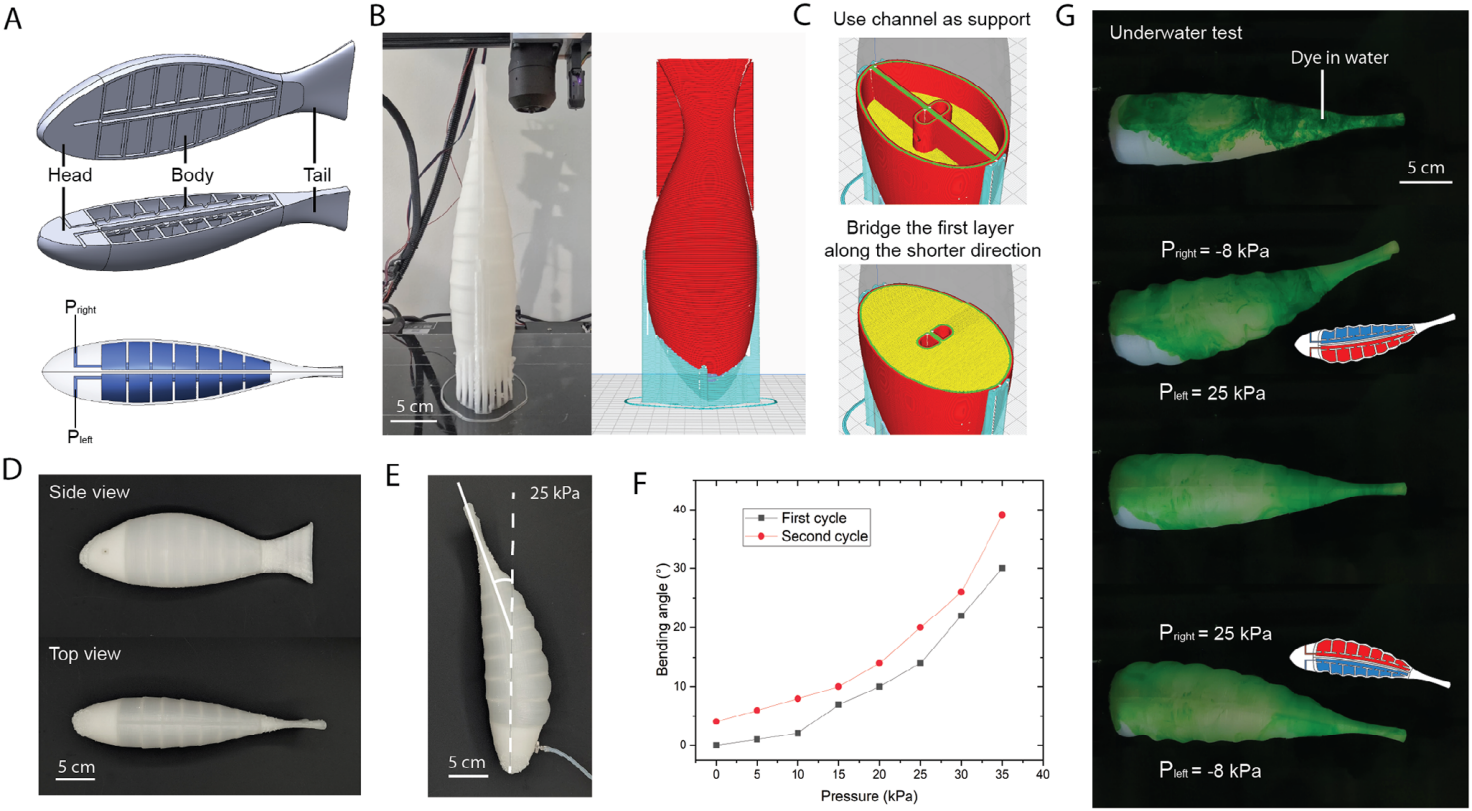
**Robot fish demonstrating airtight print for underwater applications**. (A) Cross‐sectional CAD view of the robotic fish design and schematic of the two‐chamber actuation principle. (B) Slicer view (right image) of the fish model (blue regions indicate support material) and photograph of the printed part (left image) in its final state. (C) Design principle for long bridges. (D) Photographs of the printed fish from side and top views. (E) Photograph of the robotic fish bending during actuation of its left chamber (right side from the viewer's perspective) at 25 kPa, with the bending angle measurement indicated. (F) Bending angle as a function of input pressure over two actuation cycles. (G) Underwater images of the actuated robotic fish demonstrating full airtightness and periodic tail‐swinging motion.

We fabricated a soft robotic fish using FGF to evaluate the airtightness and structural integrity of monolithically printed components for underwater applications. The design was intentionally developed to demonstrate the feasibility of printing tall, geometrically complex structures with extended bridges and overhangs. Such features remain difficult to achieve with conventional FDM‐fabricated soft systems, which are generally constrained to low‐profile architectures. The robot consists of three sections: head, body, and tail (Figure 6A). They can be printed using 22A TPS either as separate parts assembled post‐printing or as a single monolithic structure with support material. We completed monolithic printing with support material in 17 hours and 9 minutes (Figure 6B, Movie S4, Supporting Information). The body section houses all pneumatic chambers, featuring 2 mm‐thick outer walls (four perimeters) and bridges exceeding 10 mm in length. A central spinal structure was added to distribute airflow and support bridges during printing. To enhance print reliability, all first‐layer bridges were oriented along their shorter span, and travel speed was reduced to 100 mm/s to limit vibration, especially when printing taller sections (Figure 6C). The actuator architecture comprises two independent pneumatic chambers located on the left and right sides of the body. Differential pressurization of these chambers induces lateral bending of the body and tail. The bending angle at identical input pressures increased after the first activation cycle, consistent with Mullins‐type stress softening (Figure 6E, F). During underwater testing, the robotic fish remained airtight throughout repeated actuation cycles (Figure 6G, Movie S5, Supporting Information).

### 6.3 Wearable Pressure Cuff

**FIGURE 7:**
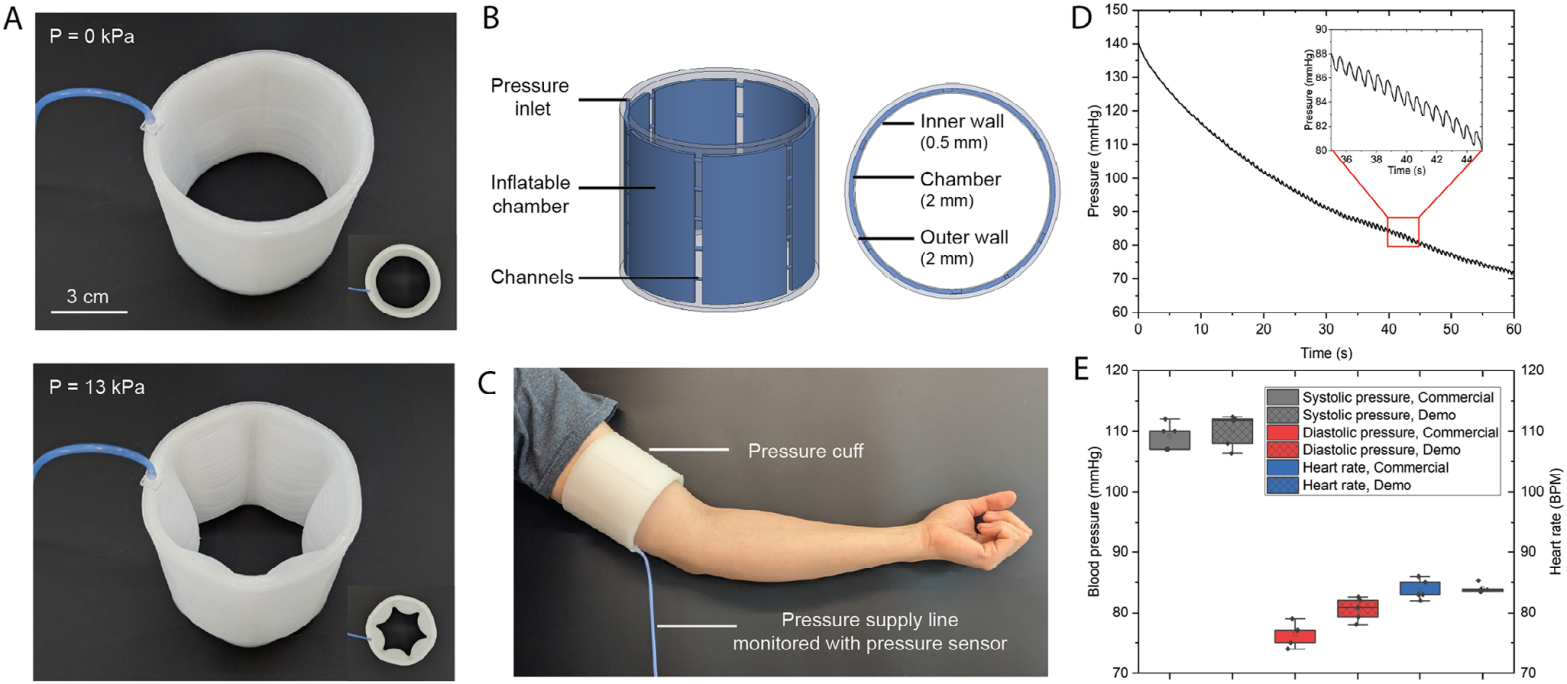
**Pressure cuff demonstrating wearable applications**. (A) Photographs of the printed cuff in unpressurized (0 kPa) and pressurized (13 kPa) states. (B) CAD model and cross‐sectional view of the pressure cuff, with blue regions showing the internal inflatable chambers and channels. (C) Demonstration of the cuff worn on an arm. (D) Pressure profile during deflation, with inset showing high‐frequency pressure fluctuations from the heartbeat. (E) Box plots of five repeated measurements of systolic pressure, diastolic pressure, and heart rate obtained using a commercial blood pressure monitor and the FGF‐printed demonstration device. For each measurement, the two cuffs were placed on opposite arms of the same subject and operated simultaneously.

We demonstrated the fabrication of wearable pneumatic devices by 3D printing a soft pressure cuff with embedded inflatable chambers using 50A TPS pellets. The design featured a single‐layer thin inner wall and a thicker outer wall, with interconnected chambers enabling uniform pressurization (Figure 7A). The cuff was printed monolithically in 9 hours and 26 minutes. Upon inflation through a single inlet, the asymmetric wall geometry caused the thinner inner wall to bulge inward, generating radial compression around the limb (Figure 7B). The geometry was optimized to operate within standard blood pressure ranges (11.3–17.3 kPa), providing a balance between compliance and stiffness. Compared to the 22A pellets used in other demonstrations, the 50A material offered improved mechanical stability while maintaining sensitivity to modest pressure variations. Despite its thin‐wall design, the cuff remained fully airtight, underscoring the reliability of the FGF extrusion process. When applied to the upper arm, the device generated uniform radial pressure and enabled blood pressure measurement via an external pressure sensor (Figure 7C,D). With five repeated measurements of systolic pressure, diastolic pressure, and heart rate, the FGF‐printed cuff exhibited comparable performance to a commercial blood pressure monitor, with good repeatability (Figure 7E). For each measurement, the cuff was first inflated to approximately 18 kPa, after which pressure data were collected during the controlled deflation phase. Although the TPS material exhibits stress softening associated with the Mullins effect, this phenomenon primarily occurs during the initial loading cycle. By applying a brief pre‐conditioning step prior to measurement, no noticeable influence of material hysteresis on pressure sensing accuracy or repeatability was observed during the tests.

## 7 Discussion

### 7.1 Positioning of FGF Among Additive Manufacturing Methods

This study investigated the practical capabilities and limitations of Fused Granulate Fabrication (FGF) for soft robotic devices and positions it within a distinct regime of additive manufacturing. High resolution resin and powder‐based processes, such as SLA, MJ, and SLS, offer high geometric fidelity but rely on expensive materials, high system costs, and post processing steps. Direct ink writing enables broad formulation flexibility but typically requires custom material preparation and rheological optimization. In contrast, FGF directly utilizes commercially available thermoplastic pellets from established industrial supply chains, enabling material access without reformulation.

Compared to conventional fused filament fabrication, FFF, pellet‐based extrusion provides improved reliability when printing soft materials. Filament systems are constrained to pre‐compounded and preselected intermediate formats by filament manufacturers and are prone to buckling and feeding instability when processing low‐Shore elastomers. The screw‐driven extrusion mechanism in FGF decouples material transport from filament stiffness, enabling stable extrusion of silicone‐soft thermoplastics at high volumetric flow rates. Pellet feedstock also reduces material cost relative to filament formats and eliminates the need for filament fabrication.

Using commercially available print heads, gantries, and off‐the‐shelf materials, this study demonstrates that FGF can serve as a practical platform for rapid prototyping of fluidically driven soft robotic devices through targeted hardware refinement, process tuning, and material‐informed selection. These advances preserve the high‐throughput nature of pellet extrusion and enable long‐duration fabrication (up to 24 h in this study) of large‐scale, airtight soft systems.

### 7.2 Material Screening for Oozing Control

One of the dominant defects in pellet‐based extrusion is stringing caused by residual melt flow after screw rotation ceases. Prior efforts have often focused on hardware‐level mitigation strategies, including cooling zones, mechanical shut‐off mechanisms, and nozzle‐level flow control. In this study, we demonstrate that material selection provides a complementary and scalable pathway for controlling residual flow.

Commercial elastomer pellets are available in thousands of formulations, often with similar Shore hardness but distinct rheological and viscoelastic characteristics. Rather than compensating for oozing through hardware complexity, we systematically identified formulations that inherently suppress residual flow while maintaining high extrusion throughput. We characterized oozing behavior across thermoplastic elastomers spanning Shore hardness values from 6A to 63A and defined material‐dependent operating windows by integrating extrusion throughput measurements, transient and long duration oozing analysis, and rheological characterization. The results show that extrusion stability is governed by the combined influence of low shear viscosity, shear thinning behavior, viscoelastic relaxation, and elastic modulus. Materials with comparable softness can exhibit markedly different oozing performance, making material choice a decisive control variable.

The relationships identified here are empirical and derived from a representative set of commercially available thermoplastic elastomers. Certain oozing metrics remain system‐dependent and should be adjusted for different printer configurations and application requirements. This work establishes a practical workflow for screening low‐oozing pellets within the broad industrial material landscape, reducing reliance on hardware modification and enabling reliable pellet‐based fabrication through informed material selection.

### 7.3 Print Quality, Mechanical Performance, and Mullins Effect

Beyond processability, this study examined print quality, mechanical properties, and bending performance of pneumatic actuators fabricated from materials spanning Shore hardness values from 6A to 50A. Stiffer materials exhibited improved dimensional accuracy, bridging capability, and overhang performance, but required higher actuation pressures. All tested pellets displayed Mullins effect‐induced stress softening, with the largest changes occurring during the first loading cycle. Although this behavior introduces deviations from idealized material models, it did not compromise long‐term actuator durability or repeatability once mechanical conditioning was applied.

From a design perspective, the Mullins effect represents both a constraint, requiring pre‐activation prior to deployment, and a potential opportunity, as controlled stress softening may be used to tune stiffness or program mechanical response in soft robotic devices. The material origins of this behavior and the influence of polymer composition require further study.

### 7.4 Limitations and Future Directions

Despite the demonstrated reliability of FGF for fabricating multiple large‐scale, airtight soft robotic systems, several limitations remain. Reduced surface roughness after support removal, localized flow inconsistencies at high flow rate, and slight geometry deviations in tall prints were observed. Addressing these challenges will require further process level studies and tighter integration of hardware optimization, material characterization, extrusion control, and geometric design. The largely unexplored landscape of commercially available thermoplastic pellets, including conductive, biodegradable, recycled, and functionalized formulations, presents substantial opportunities to expand the capabilities of FGF in soft robotics. Systematic expansion of the material dataset, integration of multimaterial extrusion and sacrificial support strategies, and implementation of closed‐loop process control represent promising directions for advancing pellet‐based fabrication of soft robotic and wearable systems, including customized prosthetic devices.

## 8 Conclusion

This work demonstrates that fused granulate fabrication can serve as a robust and accessible manufacturing platform for soft robotic devices when process constraints, extrusion dynamics, and material behavior are considered in a unified approach. By showing that commercially available thermoplastic pellets can be reliably printed into large‐scale, airtight, and mechanically durable soft systems, our study bridges the gap between labor intensive molding based soft fabrication techniques and scalable digital manufacturing.

The materials screening approach presented here provides a practical foundation for expanding FGF in soft robotics while remaining compatible with commercially available printers and established polymer supply chains. Together, these results position FGF as a compelling platform for rapid prototyping and functional deployment of soft robotic and wearable systems and create a basis for future advances in closed‐loop control, multimaterial printing, and application‐specific material design.

## 9 Experimental Section

### 9.1 Pellet printer setup

We used an Ender 3 S1 Plus FFF printer as the base platform. The standard filament extruder was replaced with a Direct3D pellet extruder. To improve feeding reliability, the Direct3D pellet hopper was redesigned with steeper wall angles to enhance gravitational flow, and its internal surfaces were coated with PTFE to reduce wall friction and prevent pellet bridging. A Raspberry Pi 4 running Klipper firmware was integrated for direct process control, and slicing was performed using Cura (v5.8.1).

### 9.2 Material Preparation

All compliant raw materials used in this study were commercially sourced (Table S1, Supporting Information). The TPS pellets selected for mechanical characterization and printing were obtained from Kraiburg TPE GmbH in Germany. To prevent moisture‐related defects such as cavity formation due to water evaporation at the print nozzle, all filaments and pellets were pre‐dried in a filament dryer (Sunlu S4) at 60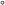 C for 6 hours and subsequently stored in a dry cabinet (Manncorp Ultra‐Dry 790V) maintained below 5% relative humidity until use.

### 9.3 Extrusion Test

For the extrusion and oozing tests, the nozzle was first preheated to the target temperature, after which the extrusion screw was rotated at a fixed speed for 60 s. Immediately after the screw stopped rotating, the extruded strand was manually removed to isolate the subsequent idle oozing behavior. The total mass of material extruded during the 60 s interval m was measured. The oozing process following screw cessation was recorded on video, and the length of the oozed filament, $l_{ooze}$, was later quantified as a function of time $t_{ooze}$. The volumetric extrusion flow rate was calculated using the measured mass m, extrusion duration t, and material density ρ as: $Q_{extrude}=m/\rho t$. Assuming that the oozed filament maintained an approximately circular cross‐section with diameter equal to the nozzle diameter d, the oozing flow rate was estimated as: $Q_{\text{ooze}}=\frac{\pi d^{2}}{4}\;\frac{\Delta l_{\text{ooze}}}{\Delta t_{\text{ooze}}}$.

### 9.4 Rheology Experiment

Rheological properties were measured using a TA Instruments HR20 rheometer equipped with an environmental test chamber and a 25 mm parallel‐plate geometry. Steady shear measurements at high shear rates were not feasible because the melts exhibited edge fracture and ejection from the plates. Therefore, oscillatory frequency sweeps were performed, and the shear‐rate–dependent viscosity was estimated using the Cox–Merz rule [54], which provides a stable and repeatable approximation for highly deformable polymer melts. For each material, a strain sweep at 100 rad/s was first performed to determine the linear viscoelastic (LVE) region. A strain amplitude of 1%, well within the LVE regime for all tested materials, was then used for oscillatory frequency sweeps. Frequency sweeps were conducted from 0.05 to 500 rad/s. Prior to testing, all samples were dehydrated at 60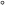 C for 6 hours, and before each measurement, samples were preheated to the target temperature for 180 s to ensure thermal equilibrium. The shear‐thinning index n was obtained by fitting a power‐law model to the viscosity data derived from the frequency sweeps. To ensure consistency across materials, the fitting was restricted to the frequency range of 10–500 rad/s, where apparent shear‐thinning behavior exhibited an approximately constant slope for all materials.

### 9.5 Tensile Test Experiment

All test bars were fabricated according to the ASTM D412 Type C standard with a thickness of 3 mm. TPE test bars were printed using either FFF or FGF, while silicone test bars were cast in molds fabricated via FFF. For the printed specimens, a single‐wall configuration was used, with infill lines oriented at 45° relative to the pull direction. Tensile tests were conducted using a universal testing machine (Instron 68TM‐50) equipped with a 500 N load cell. Following the standard, a strain rate of 500 mm/min was applied during testing. For each specimen, the initial clamp distance, width, and thickness were recorded to calculate strain and cross‐sectional area. A prestress of 0.5 N was applied for most samples, while softer materials with Shore hardness below 10 A were tested using a reduced prestress of 0.1 N.

For cycling tests, the Instron 68TM‐50 was programmed to pull the test bars between 0% strain and a specified maximum strain (varied depending on test conditions) for five consecutive cycles, followed by a final pull to a predefined maximum extension. All cyclic tests were performed at a strain rate of 500 mm/min, consistent with the uniaxial tensile tests.

### 9.6 Pneunet Test Setup

Each PneuNet was fabricated using the same print parameters as those employed for the mechanical test bars to ensure experimental consistency. The actuators were mounted on a fixed test frame against a black background, and images were captured at discrete pressure intervals. Bending angles were quantified from these images through post‐processing. For cyclic testing, the same image‐based analysis was applied to selected cycles. Actuation was controlled by an Arduino Mega, which applied 35 kPa at 5‐second intervals to achieve near‐complete bending in each cycle.

### 9.7 Simulation Setup

The bending behavior of the pneumatic actuator was analyzed using COMSOL Multiphysics 6.2. A 3D stationary study was performed with a mesh defined in normal mode and element sizes ranging from 1 to 3 mm. A fixed boundary condition was applied to the surface containing the inlet connection. To capture mechanical interactions under high pressure, contact pairs were defined between adjacent chambers using a penalty factor of 1. Gravity was included in the vertical direction to replicate experimental conditions. Internal pressurization was simulated by applying a boundary load to the inner walls of the actuator. The bending angle was quantified by extracting the normal vector components ($n_{x}$ and $n_{y}$) from the distal surface of the actuator.

Material behavior was modeled using a third‐order Ogden hyperelastic model (Supporting Information, Section S8). The model parameters for each material were fitted to true stress–strain data obtained from uniaxial tensile tests.

### 9.8 Demonstration Setup

The robotic hand comprises 15 individually controlled pneumatic actuators. Each actuator was connected to a 12V solenoid valve (0520F), driven by a MOSFET driver module (YYNMOS‐4) and controlled using an Arduino Mega 2560. The actuators in the index, middle, ring, and pinky fingers were operated at a uniform pressure of 90 kPa, while the thumb actuators were driven at a lower pressure of 15 kPa.

The robotic fish contains two internal pneumatic chambers. To maximize bending, pressure was applied to one chamber while a vacuum was simultaneously applied to the other. This differential actuation utilized four solenoid valves, two supplying positive pressure and two supplying vacuum, to achieve bending through controlled asymmetric pressurization. Both positive and negative pressures were generated by a 12V pump (SC3802PM‐A). For underwater testing, the head of the robotic fish was removed to simplify fixture mounting and ensure stable positioning during actuation (Movie S5, Figure S9, Supporting Information).

In the pressure sleeve, all chambers were connected to a single pneumatic line to enable single‐input control. A pressure regulator (AFR2000) was used to set the maximum supply pressure, and airflow was modulated by using tubing with a narrow inner diameter of 1.5 mm. Internal pressure was measured using a piezoresistive pressure sensor (MPX5050GP) connected to an analog‐to‐digital converter (ADS1115). Once the internal pressure reached a threshold of 18 kPa, the primary inflation source was deactivated. The system then switched to a high‐resistance pneumatic path, consisting of a knotted tubing segment vented to the atmosphere, to achieve a slow, controlled pressure decay suitable for oscillometric measurement. Following data acquisition, the pressure signal was processed using digital high‐pass and low‐pass filters to decouple the oscillometric pulses (heartbeats) from the deflation ramp. The Mean Arterial Pressure (MAP) was identified as the cuff pressure at the point of maximum pulse amplitude. Finally, systolic and diastolic pressures were calculated using the Maximum Amplitude Algorithm (MAA) by identifying the pressures corresponding to 55% and 85% of the peak oscillation amplitude, respectively. A clinical blood pressure monitor (Paramed) was used for comparison. However, the commercial blood pressure monitor employs proprietary oscillometric algorithms, so this comparison is intended to assess agreement and repeatability rather than replicate the internal estimation method of the reference device.

### 9.9 Statistical Analysis

All experimental data are reported as mean $\bar{x}$ ± $CI_{95\%}$ (95% confidence intervals) unless otherwise specified. The 95% confidence intervals for extrusion, oozing, and tensile tests were calculated in Python according to ${\text{CI}}_{95\%}=\bar{x}\pm t\frac{SD}{\sqrt{n}}$, where $\bar{x}$ is the sample mean, SD is the sample standard deviation, n is the sample size, and t is the two‐tailed Student's t value for a 95% confidence level with n−1 degrees of freedom. Sample sizes for each experiment are reported in the corresponding figure captions. When n<5, only the mean value is reported. No data transformation or outlier exclusion was applied unless otherwise stated.

## Conflicts of Interest

The authors declare no conflict of interest.

## Supporting information


**Supporting File 1**: advs74846‐sup‐0001‐SuppMat.pdf.

**Supporting File 2**: advs74846‐sup‐0002‐MovieS1‐S5.zip.

**Supporting File 3**: advs74846‐sup‐0003‐CADfiles.zip.

## Data Availability

The data that support the findings of this study are available from the corresponding author upon reasonable request.
