# Supplementary material for: Pellet Printing for Soft Robotic Devices
Source: Adv Sci (Weinh). 2026 Mar 23;13(30):e24237. doi: 10.1002/advs.202524237 (PMC13248815; doi:10.1002/advs.202524237)
Supplement: Supplementary file 1 — Supporting File 1: advs74846‐sup‐0001‐SuppMat.pdf. [file ADVS-13-e24237-s002.pdf]

Pellet Printing for Soft Robotic Devices

*Yijia Wu, Ju-Hung Chen, Ariana Olivares, Katherine Kostak, Stefan Pedicone, Savita Kendre, and Markus P. Nemitz**

Y. Wu, J. Chen, A. Olivares, K. Kostak, S. Pedicone, Dr. S. V. Kendre,
Prof. M. P. Nemitz
Department of Mechanical Engineering, Tufts University, Medford, MA 02155, USA
Email Address: markus.nemitz@tufts.edu

1 Material information

Table S1: The materials we used in this study, with corresponding shore hardness and manufacturers.

Material Type	Material name	Shore Hardness	Manufacturer
TPE pellets	N/A	30A	Yangzhou Baiyu
	Filaflex 60A	63A	Recreus
	TF1STL	6A	KRAIBURG TPE
	TF2STL	10A	KRAIBURG TPE
	TF2ATL	22A	KRAIBURG TPE
	TF4ATL	40A	KRAIBURG TPE
	TF5ATL	50A	KRAIBURG TPE
TPE filaments	Filaflex 60A	63A	Recreus
	Filaflex 70A	70A	Recreus
	NinjaFlex Edge	83A	NinjaTek
Silicone	Ecoflex 00-50	00-50	Smooth-On
	Dragon Skin 10 Fast	10A	Smooth-On
	Dragon Skin 20	20A	Smooth-On
	Dragon Skin 30	30A	Smooth-On

2 Additional information on system modifications

2.1 Achieving consistent extrusion

During our tests, we found that inconsistent extrusion typically results from (i) heat creep, (ii) sticky or statically charged pellets, or (iii) inconsistent pressure within the barrel.
Heat creep occurs when thermal energy from the heated barrel conducts backward along the extruder components, partially melting pellets before they reach the intended melting zone. This premature softening can cause pellets to deform or fuse together, leading to blockages or erratic feeding behavior. After analyzing infrared images from a thermal camera, we installed a high-speed cooling fan at the extruder to ensure effective thermal control and mitigate heat creep.
Pellet flow can be disrupted by surface adhesion among pellets. Pellet adhesion arises from the polymer composition, electrostatic charge accumulated during pellet handling and transport, and absorbed moisture. These effects can lead to irregular feeding, bridging, or clogging, as accumulated pellets obstruct the transport of material into the extruder barrel and prevent consistent pellet entry into the melt zone. We addressed accumulation and feeding issues by screening pellets of similar Shore hardness to identify those with inherently low surface adhesion and reduced susceptibility to static charge. We also dehumidified the pellets prior to printing to eliminate moisture-induced cohesion. On the hardware side, the pellet hopper was redesigned with a steeper wall angle to enhance gravitational flow, and the internal surfaces were coated using a polytetrafluoroethylene (PTFE) spray to reduce wall friction and mitigate pellet bridging.
After resolving hardware and material issues, we continued to observe intermittent extrusion inconsistencies. We identified the root cause as a mismatch between the commanded and actual extrusion rates. Excess flow or slow print speed caused molten material to accumulate above the nozzle, generating back pressure that led to brief over-extrusion once

a threshold was exceeded. Conversely, insufficient flow or high print speed caused under-extrusion. We resolved this issue by fine-tuning the print speed and flow rate and keeping the parameters static throughout the print to maintain a stable extrusion balance.

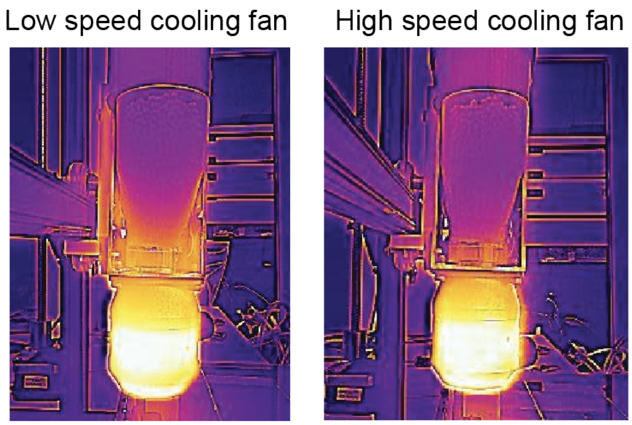

Figure S1: **Heat creep prevention.** Thermal image of the print head before and after installing a high-speed cooling fan.

2.2 List of hardware modifications

The printer was systematically modified to improve reliability and performance when printing soft TPS pellets. The key modifications are summarized below:

- A higher-speed cooling fan was installed to minimize heat creep and prevent premature softening of pellets (**Figure S1**).
- The pellet hopper was redesigned with a steeper wall angle to promote consistent gravitational flow, and printed with SLA printer to ensure smooth surface. STL file of the hopper was included as additional file.
- Internal surfaces of the hopper were coated with polytetrafluoroethylene (PTFE) spray to reduce friction and mitigate pellet bridging.
- Polypropylene tape was applied to the print bed to improve first-layer adhesion and prevent warping.
- A time-of-flight sensor was integrated to monitor pellet levels in the hopper and prevent extrusion issues due to low material volume.

3 Print parameters

3.1 FGF print parameter tuning

Because the TPS pellets were not originally formulated for 3D printing, no official print parameters are provided by the manufacturer. Instead, recommended injection molding parameters were used as a starting point. These guidelines specify cylinder temperatures for a standard 3-zone polyolefin screw, whereas the print head used in this study features a single heating zone. Consequently, the recommended final melt temperature was selected as the initial nozzle temperature, and the specified maximum temperature was adopted as the upper limit.

Print parameter tuning was conducted in two main stages. The first stage focused on establishing basic printability, defined as consistent material extrusion at practical speeds with sufficient bonding between adjacent lines and layers. Nozzle and bed temperatures were the primary variables at this stage and were typically adjusted at reduced print speeds to increase reliability, with flow rate calibrated accordingly. Calibration blocks and tensile test specimens served as evaluation structures during this phase. The second stage aimed to achieve airtight printing suitable for pneumatic devices. This required optimizing the interplay among print speed, flow rate, and temperature to ensure consistent deposition, complete layer fusion without internal voids, and minimal oozing. The primary objective was to maximize print speed without compromising airtightness. The PneuNet actuator served as the benchmark geometry for this optimization phase.

3.2 Final parameters

For each material, identical print parameters were used to fabricate both the tensile test specimens and the PneuNet actuators, ensuring that the tensile data could be directly applied to numerical simulations of the actuators (**Table S2, S3**). A fixed print speed of 30 mm/s was selected for all TPS pellets to maintain consistent print quality across materials. However, stiffer materials demonstrated compatibility with higher print speeds without compromising airtightness. Accordingly, an additional set of optimized print parameters for high-speed, airtight printing with stiffer materials is also provided (**Table S4**).

Table S2: General FGF and FDM printing parameters. *Refer to **Tables S3 and S4** for detailed extrusion parameters for pellets of different Shore hardness.

**Parameter**	**FGF**	**FDM (60A, 70A)**
Nozzle size (mm)	0.5	0.5
Layer height (mm)	0.2	0.2 (test bars)/ 0.1 (airtight parts)
Print speed (mm/s)	30	10
Infill overlap (mm)	0.2	0.25
Retraction distance (mm)	0.8	0
Extrusion multiplier	*	1.3

Table S3: FGF print parameters for TPE materials of different Shore hardnesses, used for printing tensile test bars, PneuNets, and print quality evaluations.

**Parameter**	**6A**	**10A**	**22A**	**40A**	**50A**
Print speed (mm/s)	30	30	30	30	30
Nozzle temperature (°C)	215	215	240	240	240
Bed temperature (°C)	60	60	30	30	30
Initial layer flow (%)	120	120	120	150	150
Overall flow (%)	160	160	180	320	500
Wall flow (%)	180	180	200	420	550

Table S4: High-speed FGF printing parameters for 6A–50A TPE materials. All parameter sets were validated by printing airtight PneuNet actuators.

**Parameter**	**6A**	**10A**	**22A**	**40A**	**50A**
Print speed (mm/s)	50	50	50	50	50
Nozzle temperature (°C)	215	215	240	240	240
Bed temperature (°C)	60	60	30	30	30
Initial layer flow (%)	120	120	120	150	150
Overall flow (%)	160	160	160	250	350
Wall flow (%)	180	180	180	300	380

4 Print quality evaluation

4.1 Dimensional tests

Dimensional accuracy was quantified along the X, Y, and Z directions of 20 mm calibration cube using mean signed error, root-mean-square error (RMSE), and standard deviation across 6 cubes. All lengths are measured with a caliper with caution, avoiding over-squeezing the samples. Since the print parameters are tuned to over-extrude to ensure air-tightness, the cubes are printed with 50% infill instead of 100%.

Table S5: Dimensional accuracy of printed samples along the X, Y, and Z directions relative to the nominal dimension (20 mm). Accuracy is quantified using the mean signed error (bias), root-mean-square error (RMSE), and standard deviation (SD).

**Material**	X_{bias}	X_{RMSE}	X_{SD}	Y_{bias}	Y_{RMSE}	Y_{SD}	Z_{bias}	Z_{RMSE}	Z_{SD}
50A	0.008	0.078	0.080	0.036	0.082	0.076	0.064	0.108	0.089
40A	0.103	0.135	0.089	0.008	0.077	0.079	0.070	0.109	0.088
22A	0.092	0.109	0.061	−0.017	0.075	0.075	0.072	0.113	0.090
10A	−0.367	0.379	0.096	−0.343	0.348	0.060	0.051	0.081	0.065
6A	−0.518	0.529	0.108	−0.467	0.490	0.155	0.119	0.146	0.086

4.2 Overhang and bridge tests

Printability of unsupported features was evaluated through bridging and overhang tests. Two standards were applied: critical and maximum. "Critical" represents the start of obvious visible distortion (e.g., material sagging) while still allowing successful print completion, whereas "Maximum" represents the largest bridge length or overhang angle achievable without structural failure.

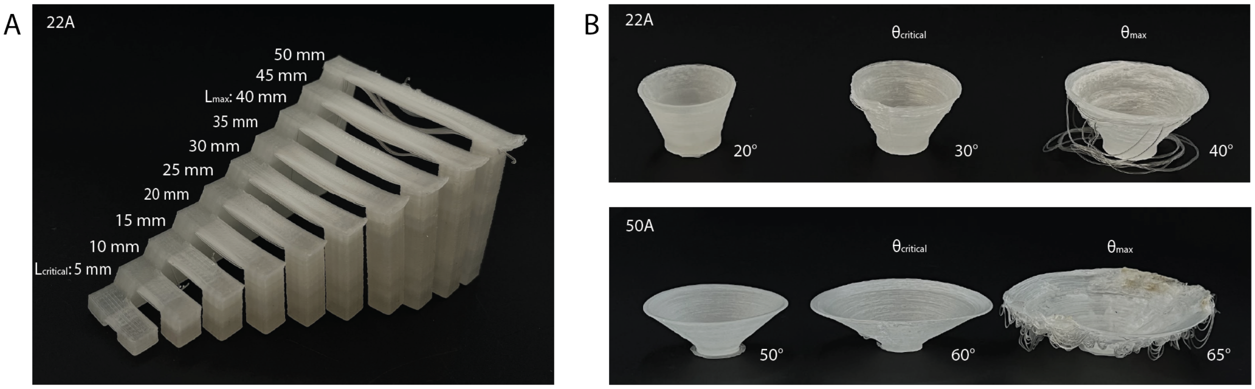

Figure S2: **Examples of bridging and overhang performance** (A) Photo of bridging test sample printed with 22A pellets with labelled critical and maximum bridge distances. Though the structure can still be printed at 40 mm, the top layers are obviously under-extruded, so it is considered the maximum bridge distance. (B) Photos of overhang test samples printed with 22A and 50A pellets. At critical overhang angles, printed cones have obvious material sagging defects. At maximum overhang angles, the cones can still be printed, but with large geometry distortion.

Table S6: Bridging and overhang performance of printed materials, evaluated by bridge length (L) and overhang angle (θ).

**Material**	$L_{critical}$ (mm)	$\theta_{critical}$ (°)	L_{max} (mm)	θ_{max} (°)
6A	3	20	25	30
10A	3	20	25	30
22A	5	30	40	40
40A	5	60	65	65
50A	5	60	70	65

4.3 Layer fusion evaluation

To assess internal print quality, cross-sectional microscopy was performed on representative printed samples. The images reveal continuous filament deposition and well-fused interfaces between adjacent strands and layers for all tested materials (**Figure SS3**), with no observable interfacial voids or delamination. These results confirm that, despite differences in dimensional accuracy and unsupported feature limits, the optimized FGF process enables robust interlayer bonding and airtight structures across a wide range of soft thermoplastic elastomers.

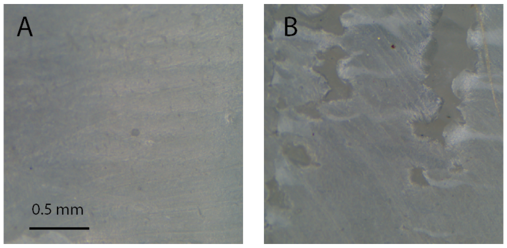

Figure S3: **Microscope images of 22A samples** (A) proper extrusion; no gaps observed. (B) under extrusion; obvious gaps observed.

5 Flow rate conversion

For the extrusion test and print parameter tuning, it is necessary to understand the relationship between print settings, volumetric flow rate, and extrusion screw rotational speed. Because each slicer and printer firmware implements this conversion differently and often includes additional hidden parameters, we present here the specific method used in this study as a general reference rather than a universal prescription.
The desired volumetric flow rate, Q_{desired}, is determined from the print settings as

$$Q_{\text{desired}} = vhd_{\text{n}}, \tag{1}$$

where v is the print speed (mm/s), h is the layer height (mm), and d_{n} is the nozzle diameter (mm). If the slicer flow factor f is set to a value other than 100%, the effective volumetric flow rate becomes

$$Q_{\text{adjusted}} = fQ_{\text{desired}}. \tag{2}$$

Most slicers and printer firmware are designed for filament-based fused filament fabrication (FFF) systems. As a result, the conversion from volumetric flow rate to extrusion motor command is based on the filament feed rate, expressed as

$$Q_{\text{adjusted}} = A_f v_f = \pi \frac{d_{\text{f}}^2}{240} v_f, \tag{3}$$

where A_f is the filament cross-sectional area, v_f is the filament feed rate (mm/min), and d_{f} is the filament diameter (mm). The feed rate v_f is explicitly written for each extrusion segment in the generated G-code.
In an FGF printer, the filament feed rate command is mapped to the rotational speed of the extrusion screw. In Klipper firmware, this relationship is defined as

$$\omega_{\text{screw}} = \frac{v_f}{d_r}, \tag{4}$$

where ω_{screw} is the extrusion screw rotational speed (RPM) and d_r (mm) is the parameter `rotation_distance`. In this study, a default value of $d_r = 7.6190$ mm, as provided in the Ender 3 printer configuration file, was used.
Combining the above relations, the desired volumetric flow rate can be related to the extrusion screw rotational speed as

$$Q_{\text{desired}} = vhd_{\text{n}} = \frac{\pi d_{\text{f}}^2 d_r}{240f}\,\omega_{\text{screw}} \tag{5}$$

6 Additional oozing and rheology tests results

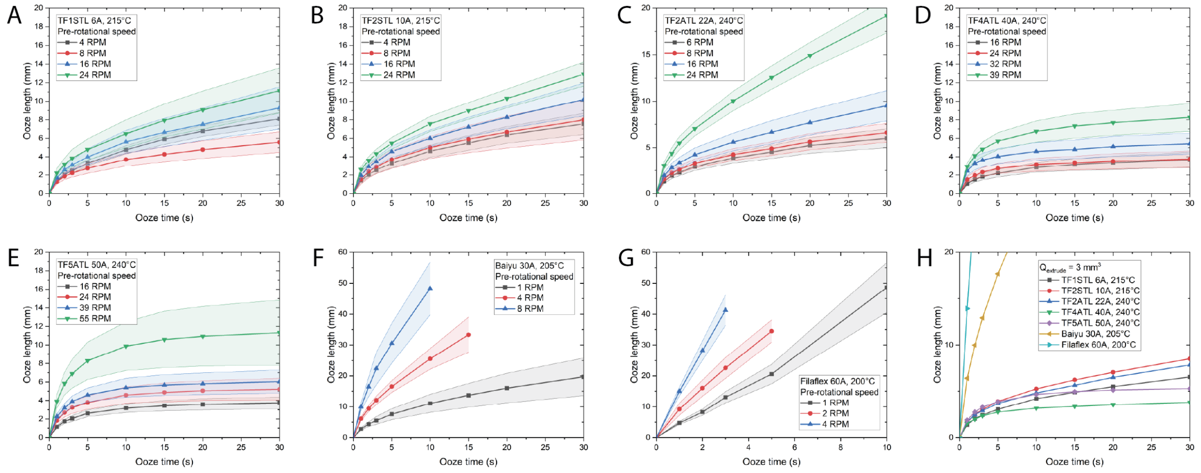

Figure S4: **Additional oozing tests results** (A) TF1STL 6A at 215°C, (B) TF2STL 10A at 215°C, (C) TF2ATL 22A at 240°C, (D) TF4ATL 40A at 240°C, (E) TF5ATL 50A at 240°C, (F) Baiyu 30A at 205°C, (G) Filaflex 60A at 200°C, (H) Oozing profile of all tested materials at an extrusion flow rate of 3 mm^3.

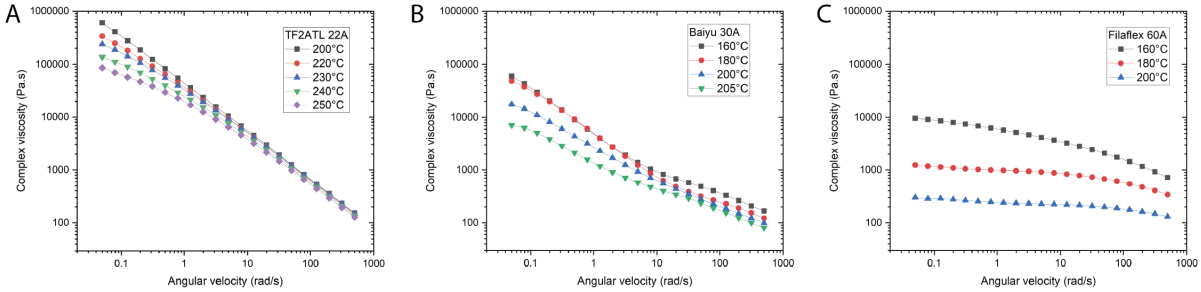

Figure S5: **Temperature-dependent rheological behavior of selected TPE pellets.** (A) TF2ATL 22A: pronounced shear-thinning behavior is preserved across the tested temperature range, while the low-shear viscosity increases at lower temperatures. (B) Baiyu 30A: both low- and high-shear viscosities vary significantly with temperature, indicating strong thermal sensitivity of melt flow behavior. (C) Filaflex 60A: relatively weak shear-thinning behavior is observed even at lower temperatures, with overall viscosity decreasing substantially as temperature increases.

Table S7: **Summary of oozing properties of the tested TPE pellets.** T_{nozzle} represents nozzle temperature. L_{2s} represents the oozed length at 2 s when the $Q_{extrude}$ is 3 mm^3. $\bar{k}$ represents the average of $Q_{ooze}/Q_{extrude}$ for all measured conditions, and $\bar{k}_{2s}$ represents the ratio at the first 2 seconds and $\bar{k}_{30s}$ represents the ratio from 15 to 30 s.

Material	T_{nozzle} (°C)	L_{2s} (mm)	$\bar{k}_{2s}$	$\bar{k}_{30s}$
TF1STL 6A	215	2.03	0.0839	0.0109
TF2STL 10A	215	2.51	0.0905	0.0119
TF2ATL 22A	240	2.46	0.0989	0.0118
TF5ATL 40A	240	1.99	0.0687	0.0024
TF5ATL 50A	240	2.75	0.0837	0.0015
Baiyu 30A	205	10.00	0.3840	/
Filaflex 60A	200	25.94	0.9281	/
Filaflex 60A	180	15.74	0.4658	/

Table S8: **Summary of rheological properties of the tested TPE pellets.** $\eta(0.05)$ represents low-shear viscosity at 0.05 rad/s. $\eta(500)$ represents high-shear viscosity at 500 rad/s. n and K represent shear thinning index and consistency index fitted by power law model. ω_c represents G'/G'' crossover frequency.

Material	T_{nozzle} (°C)	$\eta(0.05)$ (Pa·s)	$\eta(500)$ (Pa·s)	n	K	ω_c (rad/s)
TF1STL 6A	215	92533	71.98	0.1133	18498	< 0.05
TF2STL 10A	215	135091	96.66	0.1279	22233	< 0.05
TF2ATL 22A	240	137365	137.82	0.1332	31901	0.17
TF5ATL 40A	240	193437	132.69	0.1285	30268	0.07
TF5ATL 50A	240	324930	155.89	0.1344	33443	< 0.05
Baiyu 30A	205	10089	58.42	0.5893	2355	363
Filaflex 60A	200	300	136.04	0.8734	315	> 500
Filaflex 60A	180	1235	343.07	0.7779	1536	> 500

7 Additional material characterization

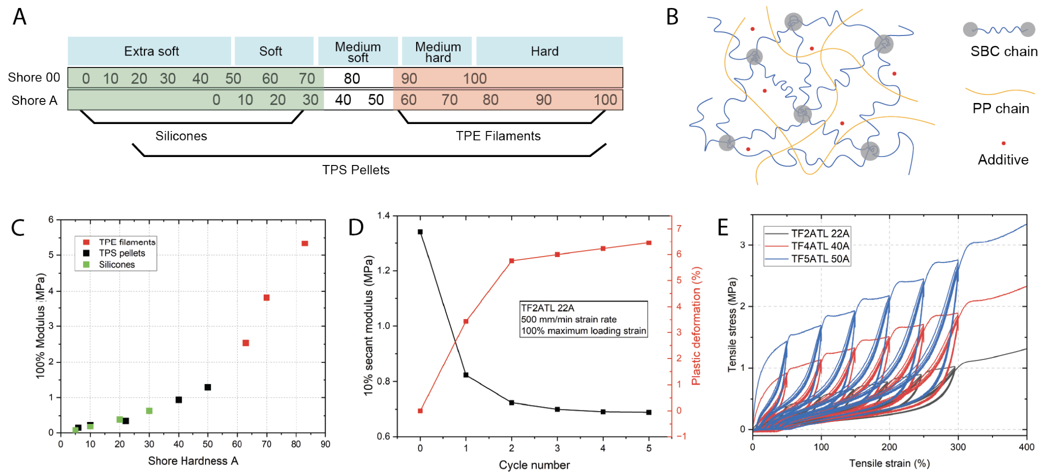

Figure S6: (A) Comparison of Shore hardnesses across commercially available silicones, TPE filaments, and TPS pellets, illustrating the broader softness range accessible through pellet-based materials. (B) Microstructure of an SBC–PP blend TPS material. PP is commonly incorporated into TPS formulations to tune elasticity and processability. (C) 100% tangent modulus versus Shore hardness, showing the stiffness–hardness correlation. (D) Evolution of 10% secant modulus and plastic deformation over five loading–unloading cycles at 100% maximum strain, illustrating Mullins effect and stabilization after the first cycle. (E) Stress-strain curves of 22A, 40A, and 50A materials undergoing the cycling test with increasing maximum maximum loading strain.

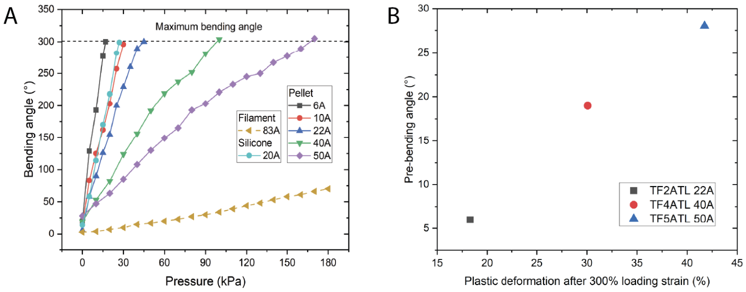

Figure S7: **Pressure–bending response of actuators fabricated from various materials.** (A) Bending angle versus input pressure for actuators printed with TPS pellets (6A, 10A, 22A, 40A, 50A), TPU filament (83A), and molded silicone (20A). Softer materials achieve larger bending angles at lower pressures. (B) Pre-bending angle versus plastic deformation after 300% maximum loading strain measured in cyclic tensile tests.

8 Hyperelastic model fitting

Uniaxial tensile tests were performed on each TPS pellet according to ASTM D412 standards for vulcanized rubbers and elastomers to characterize their mechanical properties. All specimens sustained strains exceeding 200% and exhibited large deformations comparable to silicone rubbers. Accordingly, the Ogden model was selected as the hyperelastic constitutive model, given its suitability for capturing nonlinear elastic behavior at high strains. The data fitting procedure followed a standard hyperelastic modeling approach [1]. In an N-order Ogden model, the strain energy density W is expressed with three principal stretches $\lambda_j, j = 1, 2, 3$

$$W(\lambda_1, \lambda_2, \lambda_3) = \sum_{p=1}^{N} \frac{\mu_p}{\alpha_p} \left(\lambda_1^{\alpha_p} + \lambda_2^{\alpha_p} + \lambda_3^{\alpha_p} - 3 \right) \tag{6}$$

where μ_p and α_p are material parameters. Under the isotropic incompressible material property and uniaxial loading assumption, the principal Cauchy stress σ_{uniax} function can be derived as

$$\sigma_{\text{uniax}} = \sum_{p=1}^{n} 2\mu_p \left[\lambda^{\alpha_p - 1} - \lambda^{-\frac{1}{2}\alpha_p - 1} \right] \tag{7}$$

To estimate the Ogden coefficients (μ_p, α_p), the mean true stress–strain response from five tensile tests was first calculated. The `least_squares` function from `scipy.optimize` in Python was then used to minimize the sum of squared errors between the predicted Cauchy stress (based on the estimated coefficients) and the experimental Cauchy stress. Since the initial coefficient values can influence the optimization outcome, the standard error of the estimate S was used to evaluate the fitting performance. Ten sets of random initial coefficients were tested, and the model with the lowest S value was selected as the final result. Here, y_i denotes the experimental value, $\hat{y}_i$ the predicted value, n the number of data points, and k the number of coefficients.

$$S = \sqrt{\frac{\sum_{i=1}^{n} (y_i - \hat{y}_i)^2}{n - k - 1}} \tag{8}$$

For each material, Ogden models of first-, second-, and third-order were all fitted, and the model yielding the lowest standard error S was selected and reported in **Table S9** for use in numerical simulations.

Table S9: Fitted Ogden model parameters for materials with different Shore A hardness.

**Shore A**	μ_1 **(MPa)**	μ_2 **(MPa)**	μ_3 **(MPa)**	α_1	α_2	α_3
6	0.00036	0.04168	–	5.36505	2.27831	–
10	0.00020	0.07418	–	6.07880	2.73905	–
22	0.17570	-0.02606	-0.12322	3.79427	-9.71010	4.32167
40	-0.18264	-0.17852	0.41687	-12.42437	6.22649	2.16209
50	-0.29832	-0.30330	0.65090	6.43886	-12.85631	2.02424

9 Demonstration details

The STL files of all demos are provided as additional supporting information.

9.1 Print parameters for demos

Table S10: Print parameters for three demonstrations.

Parameter	Robotic hand (22A)	Robotic fish (22A)	Pressure cuff (50A)
Print speed (mm/s)	40	30	40
Nozzle temperature (°C)	240	240	240
Bed temperature (°C)	30	30	30
Initial layer flow (%)	100	120	150
Overall flow (%)	180	180	500
Wall flow (%)	180	200	550
Infill	50% gyroid	50% gyroid	100% zigzag

9.2 Robotic fish support generation and removal

The robotic fish required two support regions near the head and tail (**Figure S8**).
The support structure near the head was automatically generated by the slicer. Support blockers were applied to prevent support material from being generated inside the internal pneumatic channels. The support overhang angle was set to 20°, and the support–object separation distance was set to 0.4 mm. Most of the support material in this region could be removed manually by hand; however, small residual features near the tip of the head required the use of pliers to ensure complete removal. This post-processing step resulted in a slight reduction in surface roughness at the contact regions. While surface finish was not a primary focus of this study, applications requiring smoother external surfaces could benefit from manually designed supports in the CAD model or from printing the head section as a separate component.
The support structure near the tail was manually designed and incorporated directly into the CAD model. These supports were positioned 0.4 mm away from the fish body and tail, enabling easy removal by hand without the use of tools. No noticeable surface defects or channel blockages were observed after support removal.

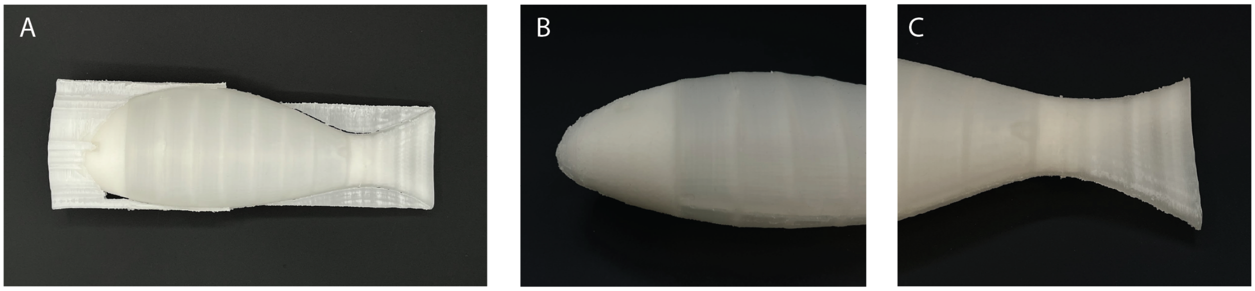

Figure S8: **Pictures of the printed robotic fish before and after support removal** (A) Picture of the fish before support removal. (B) Close-up view of the fish head after support removal. (C) Close-up view of the fish tail after support removal.

9.3 Robotic fish underwater test setup

The head segment of the soft robotic fish was removed from printing to simplify mounting. The printed body and tail were mounted to a submerged test fixture about 10 cm underwater, and the pneumatic inlet tubing was directly connected to the body to provide the pressure input required for bending motion (**Figure S9**). A green background was placed behind the water tank. During final imaging, ambient lights were turned off to minimize reflections on the tank surface, and the camera was zoomed in to capture only the region surrounding the fish.

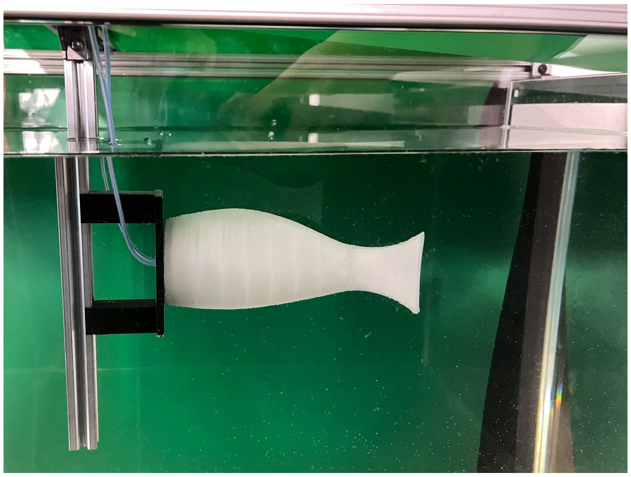

Figure S9: **Picture of the side view of the robotic fish underwater test setup.**

References

[1] L. Marechal, P. Balland, L. Lindenroth, F. Petrou, C. Kontovounisios, F. Bello, *Soft Robotics* **2021**, *8*, 3 284.
